# Hippocampal Discoveries: Spatial View Cells, Connectivity, and Computations for Memory and Navigation, in Primates Including Humans

**DOI:** 10.1002/hipo.23666

**Published:** 2024-12-17

**Authors:** Edmund T. Rolls

**Affiliations:** ^1^ Oxford Centre for Computational Neuroscience Oxford UK; ^2^ Department of Computer Science University of Warwick Coventry UK

**Keywords:** attractor network, consolidation, episodic memory, hippocampus, memory recall, navigation, neocortical memory

## Abstract

Two key series of discoveries about the hippocampus are described. One is the discovery of hippocampal spatial view cells in primates. This discovery opens the way to a much better understanding of human episodic memory, for episodic memory prototypically involves a memory of where people or objects or rewards have been seen in locations “out there” which could never be implemented by the place cells that encode the location of a rat or mouse. Further, spatial view cells are valuable for navigation using vision and viewed landmarks, and provide for much richer, vision‐based, navigation than the place to place self‐motion update performed by rats and mice who live in dark underground tunnels. Spatial view cells thus offer a revolution in our understanding of the functions of the hippocampus in memory and navigation in humans and other primates with well‐developed foveate vision. The second discovery describes a computational theory of the hippocampal‐neocortical memory system that includes the only quantitative theory of how information is recalled from the hippocampus to the neocortex. It is shown how foundations for this research were the discovery of reward neurons for food reward, and non‐reward, in the primate orbitofrontal cortex, and representations of value including of monetary value in the human orbitofrontal cortex; and the discovery of face identity and face expression cells in the primate inferior temporal visual cortex and how they represent transform‐invariant information. This research illustrates how in order to understand a brain computation, a whole series of integrated interdisciplinary discoveries is needed to build a theory of the operation of each neural system.

## 1 Introduction

The aim of this article is to provide insights into how scientific discoveries are made, and to place those discoveries in the context of advances in understanding our brains, taking my research on the hippocampus and related systems as an example. It is hoped that this will be of interest to all of those aiming to make discoveries about our brains and how they work. What is described here and elsewhere (Rolls 2023c) shows how whole series of discoveries can be fundamental in order to accumulate evidence of what is represented in different brain systems and in different parts of brain systems, which is essential for understanding the computational functions performed by different brain regions (Rolls 2023c), though awards for research are often given for single discoveries.

It is important to acknowledge that discoveries in the neurosciences typically involve a research group, and often benefit from interdisciplinary collaboration. I therefore wish to acknowledge all of my collaborators who have been so important in the discoveries of my research group. Their names will be evident from the authors on each paper. In addition, I will aim to highlight some of my interdisciplinary collaborations, as they are so important for making substantial advances in understanding systems as complex as our brains. Most of my research has been on nonhuman primates or humans, because I read preclinical medicine at Cambridge, had a vocation for medicine, and wanted my research to be relevant to advancing understanding of the human brain in health and disease, including mental disorders.

## 2 Background to the Hippocampal Discoveries by Rolls' Research Group

This section shows some of the thinking that led to the discoveries on the hippocampus described in Section 2. It illustrates some of the thinking behind how discoveries can be made in neuroscience.

### 2.1 The Discovery of Primate Lateral Hypothalamic Neurons Responding to the Sight and Taste of Food Reward, and Mediating Brain‐Stimulation Reward

One of my first interests in the neurosciences was brain systems involved in reward and emotion (Rolls 2014a). I extended the work on self‐stimulation by Olds and Milner (1954) in the rat, and discovered that brain systems involved in brain‐stimulation reward in primates as well as rodents include the lateral hypothalamus and structures along the medial forebrain bundle, the orbitofrontal cortex, and the amygdala, and was able to show by recording from single neurons that stimulation at any of these brain sites activated neurons in all of these brain sites (Rolls 1974, 2005). The discovery on the role of the orbitofrontal cortex in brain‐stimulation reward was foundational for later discoveries on the functions of the orbitofrontal cortex in reward, non‐reward, emotion, and their disorders including depression (Rolls 2019a; Rolls, Vatansever, et al. 2020; Rolls 2023d; Zhang et al. 2024).

But then I realized that to advance understanding it would be important to investigate whether these neurons activated from brain‐stimulation reward sites are involved in natural rewards, such as the taste of food, for that offered an avenue to advance our understanding of brain systems involved in food reward, obesity, and potentially other types of rewarded/emotional behavior (Rolls 2016c). So I set up to record from single neurons in the brain of behaving primates. We soon found that neurons in the lateral hypothalamus activated from brain‐stimulation reward sites could also in many cases be activated by the taste of food, a specific natural reward, providing an explanation for why brain‐stimulation reward of some sites occurs (Burton, Rolls, and Mora 1976; Rolls, Burton, and Mora 1980).

But on one occasion during such an experiment on taste reward I pulled a peanut out of my lab coat, and offered it to the primate for being so cooperative in the investigations. I was amazed to find that the taste reward neuron had a very large increase in firing rate to the sight of the peanut, even before the peanut was close enough for the monkey to reach out for the peanut, place the peanut in its mouth, and eat it. I then repeated this three times, and each time the neuron responded to the sight of the peanut with a large increase of firing rate. I checked the firing rate values being printed out on the ASR33 teletype by the PDP11 computer which I had programmed in Fortran, and found that the lateral hypothalamic neuron was indeed responding to the sight of food (Rolls, Burton, and Mora 1976; Rolls, Sanghera, and Roper‐Hall 1979). We performed many investigations that showed that these were food reward neurons, by showing that they gradually stopped responding when the food reward was devalued by feeding to satiety (Burton, Rolls, and Mora 1976); and that they would learn to respond to a stimulus such as the sight of a triangle if it signaled taste reward in a visual discrimination task, and would reverse its response in one trial if another stimulus such as the sight of a square was associated with food reward, and would stop responding to the triangle if it was now in the reversal associated with very mildly aversive salt solution (Mora, Rolls, and Burton 1976). We had discovered neurons that responded to the sight and usually also the taste of food, and that represented the reward value of the food (see Rolls 2014b; Rolls 2023c).

While performing these experiments in which the macaques were fed to satiety and a lateral hypothalamic food reward neuron stopped responding to the sight of food such as fruit juice, I one day after the monkey was satiated with fruit juice offered the monkey a peanut, and was amazed to see a large response from the lateral hypothalamic neuron to the different food (Rolls et al. 1986). I fed the macaque to satiety with peanuts, and the neuron gradually stopped responding to the sight of a peanut. But then I offered another food, a piece of banana, and discovered that the neuron, still unresponsive to the fruit juice and peanut, now responded to the sight of the banana, which the monkey readily accepted and ate with relish. I had discovered sensory‐specific satiety, and the effects that a variety of food could have in increasing food intake, at least in a single meal (Rolls et al. 1986; Rolls 2016c). I then organized some practical classes at Oxford for undergraduate students, and we were able to replicate these sensory‐specific satiety effects and the role of variety in food intake in Oxford undergraduates, at the behavioral and subjective (conscious) pleasantness levels. I invited my former wife, Dr. Barbara Rolls, who was working on thirst at the time, to participate in these practical classes, and she became interested, and collaborated with me in a number of studies on sensory‐specific satiety in humans that we published together (Rolls et al. 1981b; Rolls, Rowe, et al. 1981; Rolls et al. 1981a; Rolls, Rowe, and Rolls 1982; Rolls and Rolls 1982; Rolls, Rolls, and Rowe 1983). Barbara later told me that she was subsequently awarded a prize for sensory‐specific satiety, and I was very pleased to hear about it, for sensory‐specific satiety and its associated brain mechanisms are probably the most important factors in how much food is eaten in a single meal (Rolls 2016c), have a long‐term form that was discovered in an Ethiopian refugee camp (Rolls and de Waal 1985), and can be produced in part even by smelling or tasting a food, as I showed in an investigation with Juliet Rolls (Rolls and Rolls 1997).

### 2.2 The Discovery of Primate Orbitofrontal Cortex Neurons Responding to the Sight and Taste of Food Reward, Implementing Sensory‐Specific Satiety, and Leading to a Theory of the Functions of the Orbitofrontal Cortex in Emotion and Motivation

The question then arose about the brain source of the input to the lateral hypothalamus that produced reward‐related effects in the hypothalamus. Based on neuroanatomical (Pandya and Kuypers 1969; Jones and Powell 1970) and brain lesion (Iversen and Mishkin 1970; Jones and Mishkin 1972) evidence, two possibilities were the amygdala and orbitofrontal cortex.

When we recorded in the primate amygdala, we discovered neurons that could respond to the sight and taste of food, but their responses did not often reflect the exact preferences of the monkey, the neurons were relatively slow to reverse in the reversal of a visual discrimination task, and the effects of satiety were not as clear as in the hypothalamus (Sanghera, Rolls, and Roper‐Hall 1979; Rolls 1984; Kadohisa, Rolls, and Verhagen 2005). So we tried next the orbitofrontal cortex.

When we recorded in the primate orbitofrontal cortex, we discovered neurons that could respond to the sight, taste and/or odor of food, that did often clearly reflect the preferences of the monkey, that could reverse very rapidly in one trial in a rule‐based or model‐based type of computation, and that implemented sensory‐specific satiety (Thorpe, Rolls, and Maddison 1983; Rolls and Baylis 1994; Critchley and Rolls 1996a, 1996b; Rolls, Critchley, Mason, et al. 1996; Rolls, Critchley, and Treves 1996; Kringelbach et al. 2003). We discovered the secondary taste cortex in the primate orbitofrontal cortex (Rolls, Sienkiewicz, and Yaxley 1989; Rolls, Yaxley, and Sienkiewicz 1990; Baylis, Rolls, and Baylis 1995), and that the primary taste cortex in the anterior insula encoded the identity of the 5 tastes sweet, salt, bitter, sour, and umami (glutamate), and did not encode the reward value of the taste in that the neurons continued to respond to a taste after it had been fed to satiety (Scott et al. 1986; Yaxley, Rolls, and Sienkiewicz 1988, 1990; Kadohisa, Rolls, and Verhagen 2005; Rolls 2016a).

Extending the reward system further, we discovered that the reward value and pleasantness of fat in food in the mouth is implemented by a texture channel that responds to low values of the coefficient of sliding friction (Rolls, Critchley, et al. 1999; Verhagen, Rolls, and Kadohisa 2003; De Araujo and Rolls 2004; Verhagen, Kadohisa, and Rolls 2004; Grabenhorst et al. 2010; Rolls 2010; Rolls, Mills, et al. 2018), which has many implications for the development of foods that will have the pleasant mouth feel of fat (as in, e.g., ice cream), but which need contain no fat. Extending the reward system in the orbitofrontal cortex further we discovered that olfactory and taste stimuli converge onto single neurons to implement the flavor of food, and its reward value (Rolls and Baylis 1994; Kadohisa, Rolls, and Verhagen 2005). Extending the analysis further to social types of reward, we discovered orbitofrontal cortex visual neurons that respond to face identity, or to face expression (Rolls, Critchley, et al. 2006), implicating the orbitofrontal cortex in social reward. Applying this to mental disorders, we discovered that human patients with orbitofrontal cortex damage are impaired in emotion‐related aspects of face processing in that they are impaired in identifying face expression (Hornak, Rolls, and Wade 1996; Hornak et al. 2003), and in rapid visual reward reversal tasks (Rolls et al. 1994; Berlin, Rolls, and Kischka 2004; Hornak et al. 2004), and that in autism there is disconnectivity between the face expression representation area we discovered in the cortex in the superior temporal sulcus (Hasselmo, Rolls, and Baylis 1989; Hasselmo et al. 1989; Rolls 2024b) and the orbitofrontal cortex (Cheng et al. 2015; Rolls, Zhou, et al. 2020). An implication is that in autism there are impairments in recognizing the social significant of face including facial expression stimuli, which in turn has implications for treatment by training.

Extending the analysis further, we made the discovery that an abstract type of reward, monetary reward, is represented in the human orbitofrontal cortex (O'Doherty et al. 2001; Rolls, Vatansever, et al. 2020).

These and related discoveries provided the basis for a theory of emotion and motivation in primates including humans in which emotions are states elicited by rewards or punishers or not receiving a reward, and motivational states are states in which we are trying to obtain the reward, and avoid punishers or non‐reward (Rolls 2014b, 2023d). In this theory, the primate including human orbitofrontal cortex plays a key role by receiving inputs from the ends of sensory processing systems for visual objects and faces, taste, olfaction, touch and auditory stimuli in which reward is not represented, and then converting these sensory representations to new representations in which value (reward or punisher or non‐reward) is the key part of the representation (Rolls 2023d). Outputs from this system reach language cortical regions for declarative statements about subjective pleasantness, the anterior cingulate cortex for action—goal outcome learning, the striatum for habit responses, and the ventral striatum as a route that with the habenula may allow reward to reach dopamine neurons in the midbrain (Rolls 2017, 2023d). Rolls' theory of emotion and motivation (Rolls 2014b, 2023d) has led to a theory of depression, in which the non‐reward‐related lateral orbitofrontal cortex is too responsive and has increased connectivity leading to the sad components of depression, and the medial orbitofrontal cortex is less sensitive to reward and is underconnected, leading to the anhedonia of depression (Cheng et al. 2016; Rolls 2016b, 2018; Rolls, Cheng, et al. 2018; Rolls 2019b; Rolls, Cheng, and Feng 2020; Xie et al. 2021; Zhang et al. 2024).

The neurons that we discovered in the orbitofrontal cortex that respond to visual stimuli associated with reward led me to ask where this visual input comes from, and whether reward value was represented for example in the inferior temporal (IT) visual cortex, or whether the representation there was of objects and faces, but not about their reward value. I investigated this by performing recordings from single neurons in the IT visual cortex.

### 2.3 The Discovery of Face and Object Cells With Invariant Representations in the IT Visual Cortex, That Respond Independently of Reward Value

The next question was about where the orbitofrontal cortex and amygdala receive their visual inputs, and whether the representations there were different from those in the orbitofrontal cortex and amygdala by perhaps not representing reward value. We knew that anatomically that there was a projection from V1 > V2 > V4 to the IT visual cortex (Seltzer and Pandya 1978), where some neurons with visual responses recorded under anesthesia responded to stimuli such as hands (Gross, Bender, and Rocha‐Miranda 1969; Gross, Rocha Miranda, and Bender 1972). I decided to record from IT cortex neurons in the awake behaving macaque, found much more robust neuronal responses to visual stimuli, and made the discovery (possible in the behaving animal) that IT cortex neurons represent objects but not their reward value, in that they kept responding to visual stimuli even after devaluation by feeding to satiety, and in that they did not reverse their responses to follow reward value in the reversal of a visual discrimination task (Rolls, Judge, and Sanghera 1977). That was an important discovery in enabling us to understand cortical systems‐level functional architecture in primates, for it showed that regions at the top of visual processing hierarchies represent objects and not their reward value, and that the orbitofrontal cortex is a key stage of cortical processing in representing visual (and many other) stimuli in terms of their reward and hence emotional value (Thorpe, Rolls, and Maddison 1983; Rolls 2014b, 2023c). (The systems‐level functional architecture in primates including humans is probably very different from rodents, see Chap 19 of Brain *Computations and Connectivity* (Rolls 2023c), so the study of this in primates including humans is important.)

While recording from the temporal cortical visual regions in macaques, we also discovered face neurons (Perrett, Rolls, and Caan 1979; Perrett, Rolls, and Caan 1982; Rolls 1984) (see Rolls 2011). We showed different types of face neuron in different face patches: for example, in some patches face identity is represented (in the IT cortex), and in other patches face expression (Hasselmo, Rolls, and Baylis 1986b) is represented (in the cortex in the anterior superior temporal sulcus) with face and head motion involved in social interactions also represented in the cortex in the superior temporal sulcus (Hasselmo, Rolls, and Baylis 1986a). We discovered many of the key neurocomputational properties of these face and object encoding neurons in the IT cortical visual areas: (1) Size, contrast, spatial frequency, translation, and even in some cases view invariance is represented (Rolls, Baylis, and Leonard 1985; Rolls and Baylis 1986; Rolls, Baylis, and Hasselmo 1987; Hasselmo et al. 1989; Tovee, Rolls, and Azzopardi 1994; Booth and Rolls 1998; Rolls, Aggelopoulos, and Zheng 2003), which is ideal so that when the information reaches further cortical regions such as the orbitofrontal cortex, amygdala, and hippocampus, generalization is provided across a wide range of transforms. (2) Information is represented by sparse distributed neuronal representations not by “grandmother” cells; the information increases linearly with the number of neurons for the first 10–20 most selective neurons indicating very high representational capacity; much of the information can be read in 20 ms, with the order of the spikes not informative; and the representation is weakly ergodic in terms of how information is represented by a single neuron over a long time versus by a population of neurons over a short time (Tovee et al. 1993; Tovee and Rolls 1995; Rolls, Treves, and Tovee 1997; Rolls et al. 1997; Rolls, Tovee, and Panzeri 1999; Treves et al. 1999; Aggelopoulos, Franco, and Rolls 2005; Rolls, Franco, et al. 2006; Franco et al. 2007; Rolls and Treves 2011). (3) In complex natural scenes, translation invariance is greatly reduced to about the size of objects, so that the output of the temporal cortical visual regions can be easily interpreted by receiving structures as it is primarily about one object (Trappenberg, Rolls, and Stringer 2002; Rolls, Aggelopoulos, and Zheng 2003). (4) In complex natural scenes, there is some information about the location of each stimulus relative to the fovea (Aggelopoulos and Rolls 2005), which may facilitate scene processing. (5) The pathways in the ventrolateral cortical visual “What” stream to the IT cortex have been followed using effective connectivity (Rolls et al. 2023a; Rolls, Deco, Zhang, and Feng, 2023; Rolls 2024b; Rolls and Turova 2025), and it has been shown exactly which regions respond best to faces, versus body parts, versus tools, versus scenes (Rolls, Feng, and Zhang 2024). An interesting discovery here is that even stationary visual stimuli can activate cortical areas that relate to the semantics of each stimulus type (Rolls, Feng, and Zhang 2024). For example, the sight of stationary tools and body parts can activate visual motion areas such as FST and parts of the inferior parietal cortex implicated in performing actions in space (Rolls, Feng, and Zhang 2024).

These empirical discoveries are complemented by a biologically plausible theory of how these invariant representations are set up by self‐organizing learning in the hierarchically organized cortical visual pathways from V1 to the IT visual cortex using a short‐term memory trace associative learning rule to capture the statistics of the environment (Rolls 1992; Wallis and Rolls 1997; Rolls 2012; Rolls 2021b, 2023c).

These discoveries of face cells and their properties have been followed‐up (Desimone et al. 1984; Desimone and Ungerleider 1989; Desimone 1991; Kanwisher, McDermott, and Chun 1997; DiCarlo and Maunsell 2000; Tsao et al. 2006; Tsao et al. 2008; Freiwald, Tsao, and Livingstone 2009; Liu, Harris, and Kanwisher 2010; Rust and DiCarlo 2010; Li and DiCarlo 2012; Afraz, Yamins, and DiCarlo 2014; Tsao 2014; Deen et al. 2015; Grimaldi, Saleem, and Tsao 2016; Chang and Tsao 2017; Freiwald 2020; Hesse and Tsao 2020), including by Tsao, Freiwald, and Kanwisher, who were awarded the Kavli prize in 2024 “for the discovery of a highly localized and specialized system for representation of faces in human and nonhuman primate neocortex,” and many congratulations are due to them for this award. In addition, the discovery of neurons in the cortex in the superior temporal sulcus that respond to socially relevant aspects of face stimuli such as expression and movement (Hasselmo, Rolls, and Baylis 1989; Hasselmo et al. 1989) has now been followed‐up and accepted as a third visual cortical pathway (Pitcher and Ungerleider 2021). Moreover, I have proposed that a disconnection of these face neurons that respond to face expression and gesture from the orbitofrontal cortex emotion system may be important in understanding and treating autism (Cheng et al. 2015; Rolls, Zhou, et al. 2020).

## 3 The Discovery of Hippocampal Spatial View Cells for Memory and Navigation in Primates Including Humans, and of Their Ventromedial Visual Cortical “Where” Stream

The background to this research in Section 2 shows that we had made fundamental discoveries of the representation of visual information in the primate temporal lobe cortical visual regions, and of differences in the visual representations in brain regions to which the temporal cortical visual areas project, including the orbitofrontal cortex, amygdala, and tail of the caudate nucleus (see Rolls 2023c).

There was, in addition, evidence for visual pathways to the hippocampal system (Van Hoesen and Pandya 1975; Van Hoesen 1982), which was implicated in memory in humans (Scoville and Milner 1957; Smith and Milner 1981; Zola‐Morgan, Squire, and Amaral 1986). The hippocampal memory system was thus the next natural region to investigate, I thought, in order to build up a comprehensive understanding of the functions and computations performed by another output from the temporal lobe visual cortical regions, and that was potentially very important to understand, as the hippocampal system seemed so important in episodic memory.

### 3.1 Object‐Spatial Location Neurons Discovered in Macaques

We did not know when we started this research (see Rolls 1987) what we would discover for primate hippocampal neurons. One hypothesis at the time was that the hippocampus is involved in recognition memory, but we found that a recognition memory task engaged very few primate hippocampal neurons (Rolls et al. 1993), and it was shown that the perirhinal cortex is instead a key cortical region in recognition memory (Buckley and Gaffan 2000; Buckley 2005; Waters, Basile, and Murray 2023; Murray 2024). We also knew that place cells, that respond when the rat is located at one place in an environment, were found in the rat hippocampus (O'Keefe and Dostrovsky 1971; O'Keefe 1979). We also knew that hippocampal/fornix lesions in macaques could impair tasks about spatially directed movements (Rupniak and Gaffan 1987), and that primate hippocampal neurons become engaged in a delayed spatial response task (Watanabe and Niki 1985). Taking those findings as a starting point, we set up fundamental investigations to investigate whether hippocampal neurons were coding for the spatial aspects of the response, or the spatial aspects of the stimuli that were shown before the delay period on the left or right to be remembered in this previous research. When we recorded from neurons in the monkey hippocampus in a conditional spatial response task, we found neurons related to the responses (Cahusac, Miyashita, and Rolls 1989; Miyashita et al. 1989; Cahusac et al. 1993), which was later confirmed in a paper in Science (Wirth et al. 2003).

In the object‐place memory task to assess whether the location of the stimulus was being encoded by hippocampal neurons, we displayed a trial‐unique object on the left or right of a monitor in front of the macaque (Cahusac, Miyashita, and Rolls 1989). Then during the delay period the macaque had to remember the location and identity of the stimulus. At the end of the delay period, a stimulus was shown in the left or right locations, and the macaque had to press the location if the stimulus object and the location were the same as before the delay to obtain a taste reward. Thus, the task could only be solved by remembering both the identity of the object and the location in which it was shown. It was found that some macaque hippocampal neurons respond differently for stimuli shown in different positions in space, and some respond differently when the monkey is remembering different positions in space. In addition, some of the neurons responded to a combination of object and place information, in that they responded only to a novel object in a particular place (Cahusac, Miyashita, and Rolls 1989). The abstract stated that this was the first demonstration that some hippocampal neurons in the primate have activity related to the spatial position of stimuli (shown “out there” in the world), and this was a key discovery in our understanding of hippocampal function.

Remembering where trial‐unique visual stimuli have been seen before in space provides a potential model of episodic memory, and there was evidence that hippocampal system damage in macaques could impair this type of memory (Mishkin 1982; Gaffan 1987; Parkinson, Murray, and Mishkin 1988; Murray 2024). To investigate this key type of memory further, I then set up a task in which macaques had to remember whether they had seen before a particular trial‐unique image in a particular location on a viewed monitor screen in front of them (Rolls, Miyashita, et al. 1989). We very soon discovered that some primate hippocampal neurons responded differently to different viewed locations on the screen, and that some combined this spatial tuning with information about whether the object had been shown previously in that location on a screen (Rolls, Miyashita, et al. 1989). This was a key investigation that showed that locations in space “out there” are encoded by primate hippocampal neurons, and could be combined with information about what object/image was at each location, which is a model of episodic memory. The place where the macaque was located was not what was being encoded in these experiments, for the monkey was always in one place during the investigation. These neurons are thus quite unlike rodent place cells, which respond to the place where the rodent is located (O'Keefe 1979; McNaughton, Barnes, and O'Keefe 1983; McNaughton et al. 1996; Edvardsen, Bicanski, and Burgess 2020; Kubie 2024; McNaughton 2024). (When the term rodent is used here, it refers to rats and mice, the main rodent species in which hippocampal function has been investigated.)

### 3.2 Hippocampal Spatial View Cells Encode Spatial Locations “Out There” in an Allocentric, World‐Based, Spatial Coordinate Framework

The next scientific question that I thought it was important to address was the spatial coordinate framework utilized by these hippocampal and parahippocampal gyrus spatial view neurons with responses to some but not other locations on a screen that was being viewed in front of the macaque. We performed this by moving the video monitor in front of the macaque to the left or to the right, and by moving the macaque and screen to different places in the large room. We discovered that for the majority of hippocampal spatial view cells (69%), the coding was in allocentric, world based, coordinates (Feigenbaum and Rolls 1991). For 44% of the neurons the spatial field remained in the same location on the screen (e.g., top left on the screen), even though the egocentric location (relative to the macaque) had changed. For the remainder of the allocentric neurons, the position of the screen in the world‐based framework of the room was what was encoded. This was clear evidence that these spatial view neurons code in an allocentric framework, and for the majority this was the location in the space being viewed as defined by the framework of the screen (subtending 12°) that was being viewed (Feigenbaum and Rolls 1991). In addition, 10% of the neurons coded in egocentric coordinates. Overall, the discovery of an allocentric representation was very interesting, for in rodents place cells are often described as coding in an allocentric framework (O'Keefe 1979; McNaughton, Barnes, and O'Keefe 1983; McNaughton et al. 1996; Edvardsen, Bicanski, and Burgess 2020), and this was useful consistency to help understand hippocampal computations. However, what was being encoded in an allocentric framework was completely different, with the place where the rodent is located being represented in the rat hippocampus, and in the medial entorhinal cortex by grid cells (O'Keefe 1979; McNaughton, Barnes, and O'Keefe 1983; McNaughton et al. 1996; Moser, Moser, and McNaughton 2017; Edvardsen, Bicanski, and Burgess 2020); and the location “out there” in allocentric space that is being viewed by the primate (Feigenbaum and Rolls 1991; Rolls, Robertson, and Georges‐François 1997; Rolls et al. 1998; Georges‐François, Rolls, and Robertson 1999).

### 3.3 Spatial View Versus Place for Primate Hippocampal and Parahippocampal Neurons

So far, we had discovered that hippocampal and parahippocampal gyrus neurons in macaques could represent allocentric locations in space “out there” when they were being looked at (Cahusac, Miyashita, and Rolls 1989; Rolls, Miyashita, et al. 1989; Feigenbaum and Rolls 1991), and could participate in one‐trial object‐spatial location memory (Rolls, Miyashita, et al. 1989).

But that left the issue open of the extent to which the primate hippocampus might in addition represent the place where the individual was located by having place cells, and I thought it important to investigate this. To investigate the relative roles of spatial view versus place in the responses of primate hippocampal neurons, we now set up a 2 × 2 × 2 m cue controlled environment within which the macaque could be moved to different places on wheels or by a robot, and could from those places view the cues on the walls of the enclosure (Rolls and O'Mara 1995). The responses of the majority of the neurons with spatial responses responded to the location at which the macaque was looking (5.5% of the total number of neurons recorded), independently or relatively independently of the place where the macaque was located. In addition, 0.7% of the neurons had responses that were related to the place where the individual was located, and these primate hippocampal place‐related neurons were also influenced by where the individual was looking or by whole‐body motion (Rolls and O'Mara 1995).

### 3.4 Primate Spatial View Hippocampal and Parahippocampal Neurons During Free Locomotion

At about this time (1992–1995), I led a Human Frontier Science Program grant to investigate hippocampal function, with the members including Bruce McNaughton, Carol Barnes, Alessandro Treves, and Jay McClelland. Bruce McNaughton suggested that as place cells in rodents can be influenced by whether the rat can locomote, then it would be useful to know what would happen when primates could locomote. Perhaps spatial view cells would turn into place cells was an idea.

I was skeptical that this would make a great deal of difference, for primates with their foveate vision can effectively actively explore a spatial environment by moving their eyes. But I set up to record in freely moving macaques from the hippocampus. I did this by making a special lightweight primate chair that could be moved into a horizontal position and had wheels, and that allowed the macaque's four feet to be on the ground so that he could run around freely. He was able to run fast, like a small dog, with high linear and angular velocity, as shown in the videos in the Supporting Information. The head was free to move in the horizontal plane at high angular velocity, because as the animal rotated the head, the trolley moved with his head. The head did not move in the pitch and roll planes. A camera above the individual was used to measure using two LEDs on the chair, one flashing at twice the frequency of the other, the exact location and head direction of the macaque. The eye position was measured to within 2° by field coils attached to the trolley, and a scleral search coil. We could therefore monitor in continuous time exactly where the monkey was visually fixating in the environment. The environment was a large open lab space, and this was important, for many more spatial view cells were recorded in this rich spatial environment than in the cue‐controlled environment used by Rolls and O'Mara (1995). The importance of a rich spatial environment, and the ability to locomote freely in this with vestibular and proprioceptive information, and so forth, in order to obtain the best responses from many primate spatial view cells, has recently been confirmed, in that macaque spatial view cells were much better activated during active locomotion in a rich spatial environment than in a Virtual Reality model of the same environment (Yan and Mao 2024). Our recording setup enabled the macaque to walk freely in a 2 × 2 m space in the middle of the lab, and to turn as much as the individual wished (although after about 20 min we sometimes had to stop the monkey to unwind the wires going to the ceiling high above the head).

With this setup for free locomotion, we were able to record spatial view cells while the macaque actively and freely locomoted (Rolls, Robertson, and Georges‐François 1997; Robertson, Rolls, and Georges‐François 1998; Rolls et al. 1998; Georges‐François, Rolls, and Robertson 1999). When analyzing whether they responded to spatial view, or the place where the individual was located, great care was used to implement a factorial design in which all spatial views could be seen from all places in the analysis. Unless a factorial design of this type is used, some views may only be visible from some places, which would produce a confound between analyses of whether a neuron is coding for view, or place, or has some mixed selectivity. In my opinion, this type of factorial design is essential for reliable evidence about whether neurons code for spatial view, or place, or have mixed encoding (Rolls 2023a).

**FIGURE 1:**
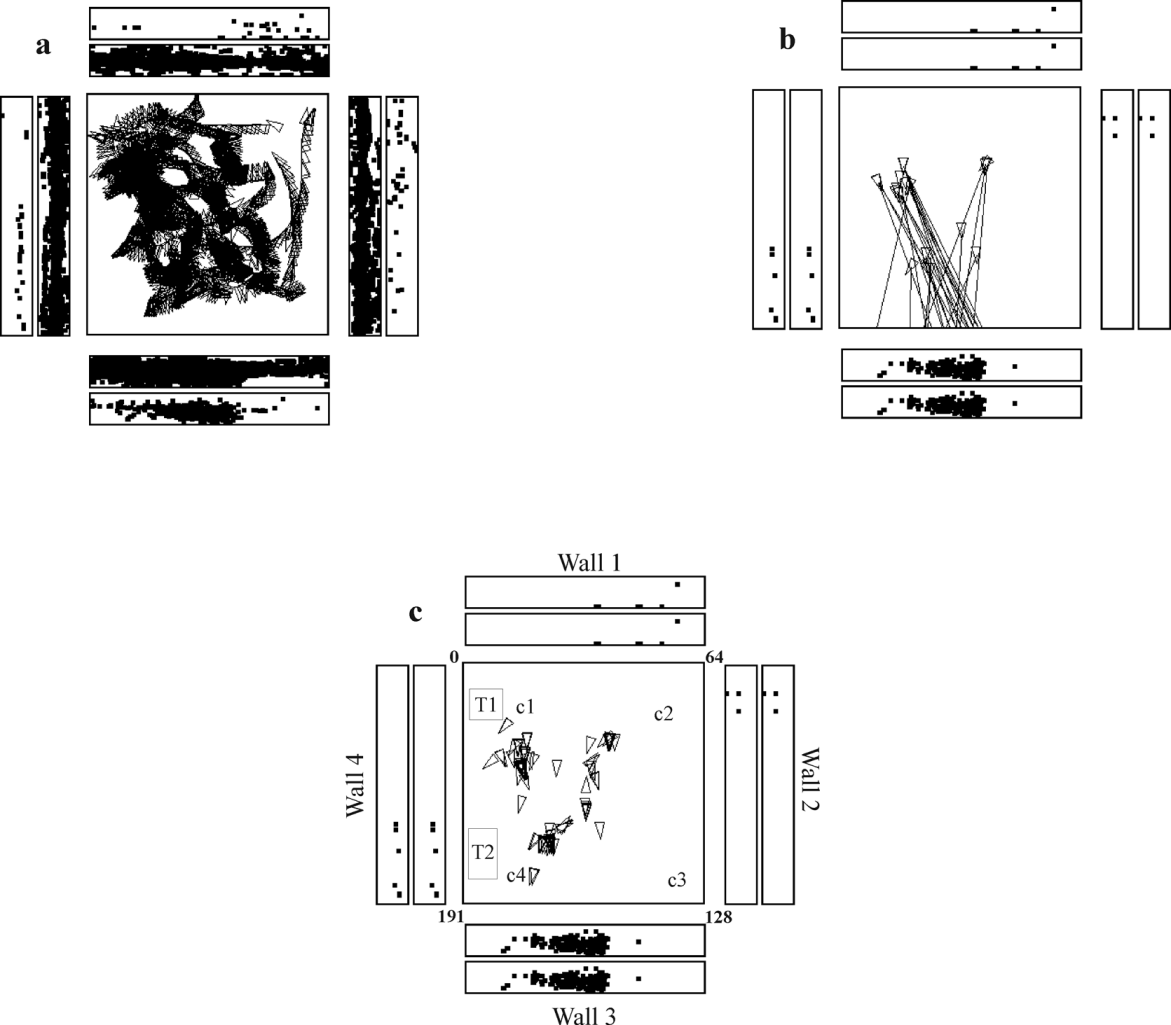
A hippocampal spatial view cell (az033) recorded while a monkey walked around in an open field area 2.5 × 2.5 m shown as the square within a rich and large laboratory environment. In (a), every time that the cell fired is shown by a spot in the outer rectangles each of which represents one of the four walls of the room. The inner rectangles show where the monkey looked on the walls. The neuron has a spatial view field on wall 3. The places to which the monkey walked are shown by the triangles, with the pointed end showing the head direction. (b) Some of the many different places at which the monkey was located when the neuron fired, and the lines show where the monkey was fixating when the spatial view cell fired. (c) Provides more evidence about the places where the monkey was located when the cell fired because he was looking at the view field on wall 3. This helps to show that the neuron responds to spatial view, and not to the place where the monkey was located. C1 to c4 are cups containing food to encourage the monkey to forage. T1 was a trolley and T2 a table. Details are provided by Georges‐François, Rolls, and Robertson (1999). Videos to illustrate the firing of spatial view neurons are described in the Data and Code Availability statement.

With this design, we were able to measure with rigorous information theoretic approaches (Rolls et al. 1998) how much information single hippocampal and parahippocampal neurons encoded about spatial view versus place during free locomotion in a rich visual environment of a laboratory. The findings were clearly that most of the information from most cells is about spatial view, and not the place where the individual is located, nor head direction, nor eye position in the head (left/right and up/down). The mutual information across locations was measured, and the mean across neurons about spatial view was 0.47 bits, about place was 0.033 bits, about head direction was 0.054 bits, and about eye position was 0.017 bits (Georges‐François, Rolls, and Robertson 1999). An example of a spatial view cell recorded during active locomotion in a macaque is shown in Figure 1, and videos to illustrate the firing are provided in the Supporting Information. Full details of these discoveries are provided elsewhere (Rolls, Robertson, and Georges‐François 1997; Robertson, Rolls, and Georges‐François 1998; Rolls et al. 1998; Georges‐François, Rolls, and Robertson 1999), and some of their implications have been described (Rolls and Wirth 2018; Rolls 2023b; Rolls 2023a). These spatial view neurons are tuned to vertical as well as horizontal locations in the viewed spatial environment (Georges‐François, Rolls, and Robertson 1999).

**FIGURE 2:**
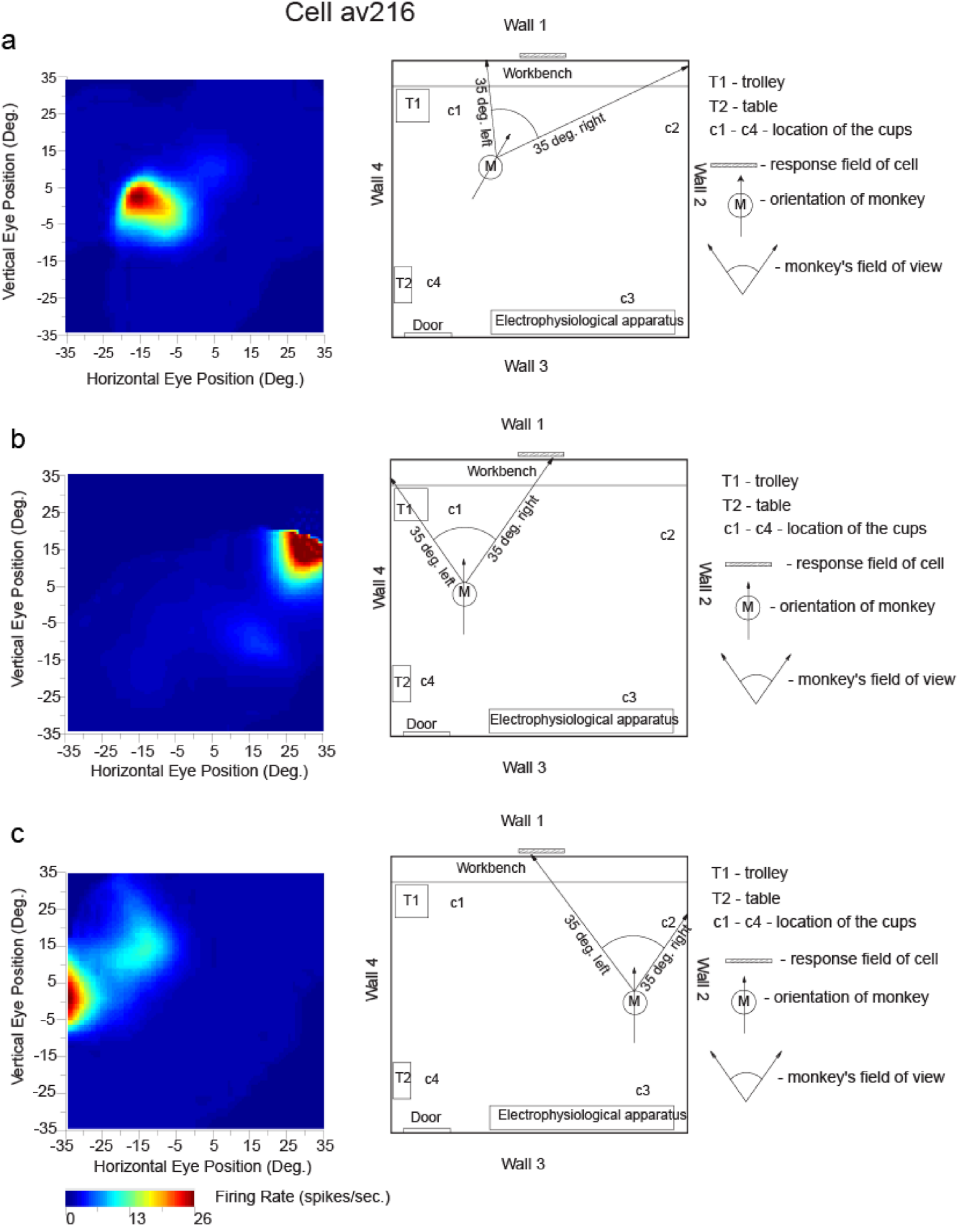
Testing of a hippocampal spatial view neuron (av216) to show that it has allocentric encoding, and that the response does not depend on where the monkey is located. The firing rate is shown as a function of the horizontal and vertical eye position, where positive values indicate right or up. The neuron responded when the monkey looked toward its view field (indicated with a hatched bar) relatively independently of place, eye position, or head direction. ANOVAs and information theory analyses performed on the same data cast in different ways confirmed this: For spatial view, the ANOVA was *p* < 0.001 with 0.217 bits in a 500 ms period for the average Shannon mutual information; for place *p* = 0.9 with 0.001 bits; for head direction *p* = 0.5 with 0.0 bits; and for eye position *p* = 0.8 with 0.006 bits (modified from Georges‐François, Rolls, and Robertson (1999)).

A feature of these discoveries is that I ensured that we could combine recordings during active locomotion in a rich open field environment in a naturalistic type of investigation, with carefully controlled complementary investigations in which the macaque was stopped. When stopped, the firing of cells was measured when the macaque was in different places, with different head directions, and with different eye positions, enabling a very clear distinction to be made that the neurons were responding to where in the environment the individual was looking, and not just the facing direction, with an example shown in Figure 2 (Rolls, Robertson, and Georges‐François 1997; Robertson, Rolls, and Georges‐François 1998; Rolls et al. 1998; Georges‐François, Rolls, and Robertson 1999). In a different investigation, visual spatial cells were confirmed in the primate hippocampus that respond to viewed locations “out there” (Mao et al. 2021). A small difference is that it was proposed that hippocampal visual spatial neurons code for the direction in which the individual is facing rather than where the macaque is looking (Mao et al. 2021), but this could be related to the fact that typically when macaques navigate, they face straight ahead in the direction in which they are most commonly looking. In situations such as this, it is helpful to perform the more controlled type of experiment illustrated in Figure 2, for this allows different head directions, places, and eye positions to be systematically explored, which as shown in Figure 2 led to the clear conclusion that it is where in the environment the macaque is looking that activates spatial view neurons (Rolls 2023b). This is of course highly adaptive, in that the location in the environment can be recognized later, even if the individual is in a some different place, and with a different head direction, which would enable a “good” (i.e., reward) at that location to be recalled, whereas it would be missed if facing direction was the encoding scheme, unless the individual was at the same place with the same head direction (Rolls 2023b). That, is, spatial view cells, because they respond allocentrically to locations out there in the environment, implement spatial representations that are invariant with respect to facing direction, head direction, and place (Georges‐François, Rolls, and Robertson 1999; Rolls 2023b). (I have discussed this with Dun Mao, the first author of the paper referred to above, and my understanding is that he accepts this point.)

### 3.5 Primate Spatial View Hippocampal and Parahippocampal Neurons Show Idiothetic (Self‐Motion) Update

I knew that rat place cells could be updated by self‐motion (McNaughton et al. 1996; McNaughton et al. 2006), and decided to test this for primate spatial view cells. This led to another key discovery about hippocampal spatial view neurons, that they can be updated idiothetically (i.e., by self‐motion) in the dark or when the view is obscured (Robertson, Rolls, and Georges‐François 1998). For example, spatial view neurons fire in the dark whenever the macaque looks toward the spatial view field. The update is based on, for example, eye movements being made by a macaque. This is idiothetic update in that there is some drift over time, and the update becomes gradually lost over 3–5 min (by which time idiothetic update in the experimenters started to fail, and they had to feel their way toward a light switch) (Robertson, Rolls, and Georges‐François 1998). This idiothetic update is not only useful for navigation when the view details are obscured (Rolls 2021a), but is also interesting as it is paralleled by idiothetic update for place in rodents (McNaughton et al. 1996; McNaughton et al. 2006), providing evidence that the rodent and primate hippocampal systems have considerable similarities computationally, but use largely different types of input, namely about place in rodents and about spatial view in primates including humans.

However, the mechanisms for path integration may be outside the hippocampus, and performed by different systems, with grid cells in the rodent medial entorhinal cortex implicated in path integration for place (Brandon et al. 2011; Giocomo, Moser, and Moser 2011; Moser, Moser, and Roudi 2014; Moser, Rowland, and Moser 2015; Moser, Moser, and McNaughton 2017), whereas the dorsal visual system of primates is implicated in transforms from retinal coordinates to head based coordinates and even to some type of allocentric encoding and in idiothetic update (Snyder et al. 1998; Rolls 2020). The implication of idiothetic update in primates is that there is a model of space and that the location in space is updated by eye movements made by the individual. Further support for this hypothesis is that primate hippocampal neurons can respond to a spatial view field toward which a macaque saccades even before the virtual reality system has updated to show the view to which the macaque is making a saccade (Wirth et al. 2017).

### 3.6 Supporting Evidence for Neurons Responding to Spatial View in the Primate Including Human Hippocampal System

Relatively recently, supporting evidence related to the discovery of spatial view neurons in primates has accumulated, with the indication that spatial view encoding is a key type of representation found in primates including humans, and that differs from what has been the primary focus in rodents, place cells.

One example is that in a virtual reality investigation of navigation in a star maze, hippocampal neurons were found that responded to where the macaque was gazing in the virtual reality space (Wirth et al. 2017). Some neurons also responded to a combination of where the macaque was and where the macaque was looking in space, though it is noted that unless all spatial views can be seen from all places in the data analyzed, the data may appear to show mixed encoding to combinations of view and place (Rolls 2023a). These neurons also show what is usually described as rate remapping (Leutgeb et al. 2005) when the chart or map (McNaughton et al. 1996; Samsonovich and McNaughton 1997; Battaglia and Treves 1998) or in this case the star maze remains unaltered, but the room cues are changed (Baraduc, Duhamel, and Wirth 2019).

Another example is that some hippocampal neurons code for the location viewed “out there” on a screen in an object‐location memory task (Chen and Naya 2020; Yang, Chen, and Naya 2023).

Another example is that in freely moving macaques, hippocampal neurons were found to respond to the spatial view toward which the macaque was looking (Mao et al. 2021). Such spatial view neurons are better activated when freely moving in a real environment than in Virtual Reality of the same environment, and are well activated when the macaque is performing an object‐viewed location task (Yan and Mao 2024).

Another example is that in marmosets freely moving in a 3D environment, approximately one‐third of hippocampal neurons responded to the view toward which the individual was looking (Piza et al. 2024).

Another example is that in a virtual reality experiment involving navigation toward goals, many macaque hippocampal neurons responded to where the animal was looking, rather than the place where the animal was located (Buffalo 2024).

Also, for humans there is now some evidence for medial temporal lobe neurons with properties like those of spatial view cells (Ekstrom et al. 2003; Miller et al. 2013). For example, in the study by Ekstrom and colleagues, some medial temporal lobe neurons were found to represent views of landmarks (Ekstrom et al. 2003). In another study of human medial temporal lobe neurons, it was found that in a Treasure Hunt game, some neurons respond to the sight of remote locations rather than the subject's own place (Tsitsiklis et al. 2020). Just like macaque spatial view cells, these neurons in humans respond when the spatial location is seen with different bearings. Also in humans some medial temporal lobe neurons reflect the learning of paired associations between views of places, and people or objects (Ison, Quian Quiroga, and Fried 2015), and this implies that views of scenes are important for human hippocampal function. Consistent with this, human functional neuroimaging studies do show hippocampal or parahippocampal activation when scenes or parts of scenes are viewed even when the human is fixed in one place for neuroimaging (Stern et al. 1996; Epstein and Kanwisher 1998; O'Keefe et al. 1998; Burgess 2008; Hassabis et al. 2009; Chadwick et al. 2010; Chadwick, Mullally, and Maguire 2013; Maguire 2014; Brown et al. 2016; Zeidman and Maguire 2016; Rolls, Feng, and Zhang 2024) (Figure 4).

The discovery of spatial view cells in primates (Cahusac, Miyashita, and Rolls 1989; Rolls, Miyashita, et al. 1989; Feigenbaum and Rolls 1991; Rolls and O'Mara 1995; Rolls, Robertson, and Georges‐François 1997; Robertson, Rolls, and Georges‐François 1998; Rolls et al. 1998; Georges‐François, Rolls, and Robertson 1999) that is leading to a revolution in understanding hippocampal function in primates including humans (Rolls 2023b; Rolls 2023a) is now therefore supported by a great deal of more recent evidence. The spatial view representations are so different from the representations provided by place cells in the rodent hippocampus of the place where the rodent is located that there are major implications for understanding hippocampal functions in primates including humans. These implications are considered in Section 3.11, but first I describe further discoveries on spatial view neurons that draw out further some of the ways in which they may be important, including how they may be important in episodic memory.

### 3.7 Hippocampal Spatial View Cells Are Involved in Object—Spatial Location Episodic Memory

The next issue that I decided to address is how the primate hippocampus is involved in an example of episodic memory, remembering where objects have been seen in scenes with rapid learning and later recall.

We discovered that in an object‐location memory task in which macaques had to rapidly learn and remember where objects were shown in a room, some hippocampal neurons (10%) responded differently to different objects independently of location; other neurons (13%) responded to the spatial view independently of which object was present at the location; and some neurons (12%) responded to a combination (conjunction, with greater responses to the combination than to each of the parts) of a particular object and the place where it was shown in the room (Rolls, Xiang, and Franco 2005). These results show that there are separate as well as combined representations of objects and their locations in space in the primate hippocampus in this type of memory task. This is a property of an episodic memory system, for which associations between objects and the places where they are seen are prototypical. Similarly, it has been shown more recently in humans that some hippocampal neurons code for the conjunction of the different elements that make up an episode (Kolibius et al. 2023).

We also discovered that in a one‐trial object—spatial location task, some hippocampal neurons responded during recall when an object cue signaled that the spatial view location should be recalled, and that other neurons responded during recall when the object was recalled by a spatial location cue (Rolls and Xiang 2006). That makes spatial view neurons highly relevant to episodic memory, and in particular to how episodic memories can be recalled by an incomplete recall cue.

### 3.8 Hippocampal Spatial View Cells Are Involved in Reward/Goal—Spatial Location Memory

The next question I addressed was whether spatial view neurons can learn where rewards, goals, are in viewed space, for that is important in both episodic memory, and in navigation toward goals. We discovered that some spatial view neurons encode the location of a high reward value in a location, and others encode the location of the lower reward value (Rolls and Xiang 2005). When the reward values are reversed, these spatial view reward neurons reverse the locations in the scene to which they respond (Rolls and Xiang 2005). What appear to be similar neurons have more recently been described that respond to the viewed location of the goal in a Treasure Hunt game (Tsitsiklis et al. 2020). Further, what may be analogous hippocampal neurons have more recently been described that respond when a chickadee looks at a particular food cache location (Chettih et al. 2024). Interestingly, these neurons in the avian hippocampus do not respond when the cache location is viewed when the food has been removed from the cache so that it is no longer a reward location, which is very analogous to the primate spatial view and reward neurons that stop responding to the high value location in a scene when it is devalued in reward reversal (Rolls and Xiang 2005). The avian evidence supports the point that good foveal vision is useful for setting up spatial view cells (De Araujo, Rolls, and Stringer 2001) (Figure 5), and also for the evidence that a random new set of CA3 neurons is chosen for each episodic memory (Rolls 2013, 2016d; Rolls and Treves 2024), termed in the chickadee a “bar code.”

The discovery of this reward‐related functionality of primate spatial view neurons (Rolls and Xiang 2005) is important, for it is very valuable to primates including humans to remember where in the environment they have seen rewards (or for that matter aversive stimuli). Interestingly, the spatial view—reward neurons are a feature of the hippocampus with its spatial (and temporal) processing, for when macaques perform an object‐reward association task, hippocampal neurons are rather unresponsive, and this type of object‐reward association learning and reversal is implemented in the primate orbitofrontal cortex (Thorpe, Rolls, and Maddison 1983; Rolls, Critchley, Mason, et al. 1996; Rolls, Vatansever, et al. 2020; Rolls 2023c).

### 3.9 A Ventromedial Cortical “Where” Visual Stream to the Human Hippocampus for Spatial View

Another key issue that I thought was important to investigate is how the primate including human hippocampus receives information about spatial view. One concept (Bicanski and Burgess 2018) has been that the dorsal “Where” visual cortical stream to the parietal lobe, which has been called a “Where” pathway (Mishkin, Ungerleider, and Macko 1983; Ungerleider and Haxby 1994), is the route for spatial information to reach the hippocampus. Another concept is that medial entorhinal cortex grid cells in rodents provide a spatial input to the hippocampus for “Where” place representations (Moser, Moser, and McNaughton 2017).

We addressed this issue by measuring effective connectivity in the human brain to analyze visual cortical processing. Effective connectivity measures the effects of each pair of brain regions in both directions on each other, and is computed in the Hopf algorithm that we use based on functional connectivity and delayed functional connectivity, with fMRI data from 171 Human Connectome Project participants in the resting state at 7 T, validated by 956 participants at 3T in the resting state, and with fMRI data from 956 Human Connectome Project participants when viewing scenes, faces, tools, and body parts (Rolls et al. 2022a; Rolls et al. 2022c; Rolls et al. 2023b; Rolls et al. 2023a; Rolls et al. 2023c, 2023d; Rolls, Wirth, et al. 2023; Rolls 2024b; Rolls, Deco, et al. 2024; Rolls, Feng, and Zhang 2024). These effective connectivity analyses are all complemented by functional connectivity and by diffusion tractography, and also included magnetoencephalography which because of its fast timecourse can provide clear evidence about the directionality of the connectivity (Rolls, Deco, Zhang, and Feng, 2023; Rolls, Yan, et al. 2024).

**FIGURE 3:**
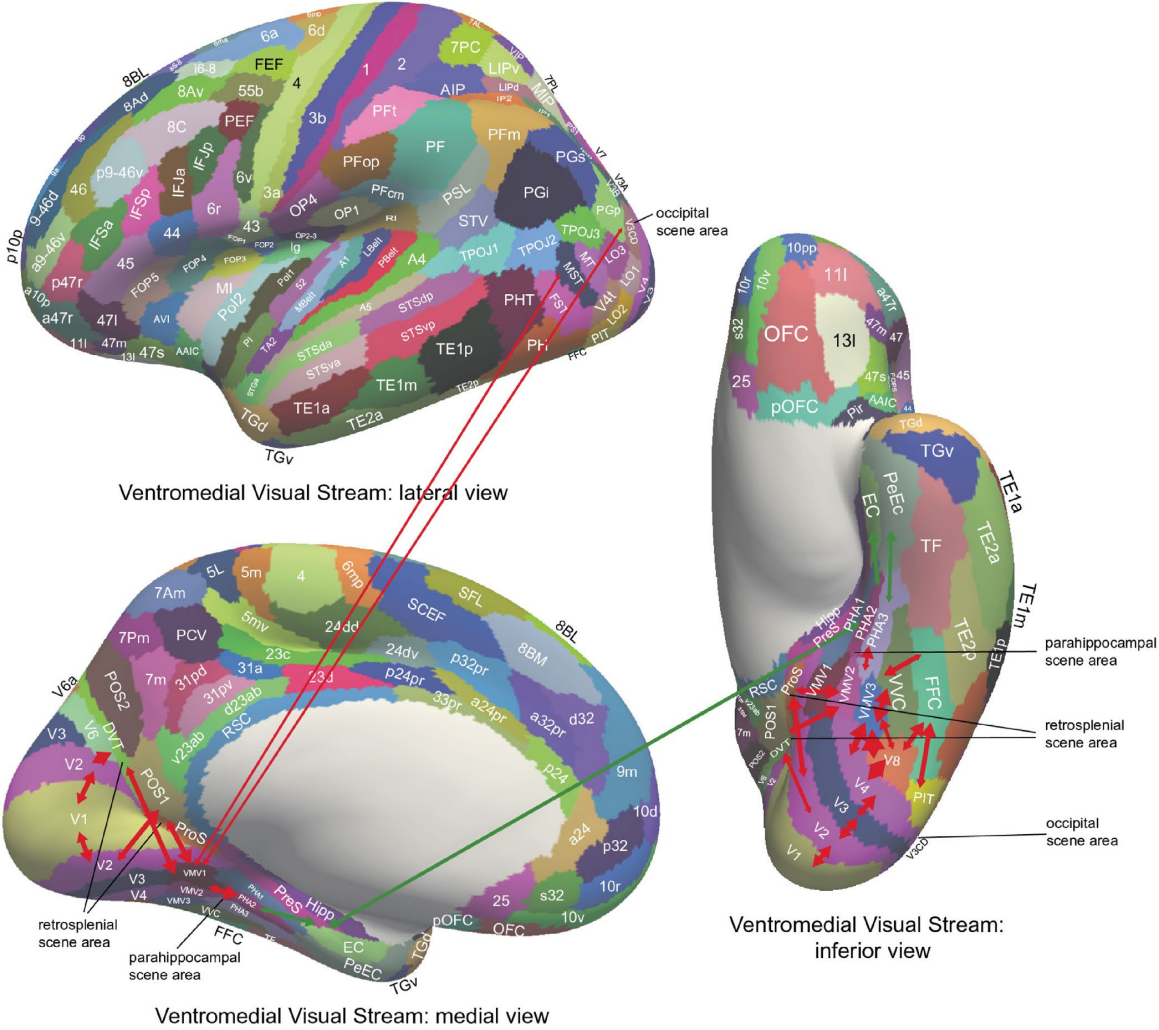
Effective connectivity of the human Ventromedial Visual Cortical Stream which reaches the parahippocampal gyrus PHA1—PHA3 regions via ventromedial (VMV) and ventral visual complex (VVC) and ProStriate regions: Schematic overview. Visual scenes are represented in the anterior parts of VMV and the posterior parts of PHA1—PHA3 (Sulpizio et al. 2020) in what is termed the Parahippocampal Place Area, PPA (Epstein and Baker 2019). The PPA might better be termed the parahippocampal scene area (PSA) as it is the scene being viewed, not the place where the individual is located, that is represented (Rolls 2023a). The retrosplenial scene area is in a band of cortex in the Prostriate cortex PRoS and Dorsal Visual Transitional cortex DVT that is posterior to region RSC (Sulpizio et al. 2020). The occipital scene area is in V3CD and borders V4 (Sulpizio et al. 2020). The green arrow shows how the ventromedial visual stream provides “where” input about locations in scenes to the hippocampal memory system from the medial parahippocampal gyrus PHA1–PHA3 region (which corresponds to TH in macaques). The connectivity from PGp to PHA regions is suggested in the text to be involved in idiothetic update of locations in scenes using a gaze direction signal. The widths of the lines and the size of the arrowheads indicate the magnitude and direction of the effective connectivity. The names of the cortical regions are from the Human Connectome Project Multimodal Parcellation (Glasser et al. 2016; Huang et al. 2022), which was used in this research (after Rolls et al. (2023a) and Rolls, Deco, Zhang, and Feng (2023)).

With these approaches, we have been able to identify a ventromedial cortical visual stream in humans illustrated in Figure 3 with connectivity from V1 and V2 to the ProStriate cortex and nearby regions where the retrosplenial scene area is that responds to scenes (Sulpizio et al. 2020; Rolls, Feng, and Zhang 2024). The ProStriate region then projects forward to ventromedial visual cortex VMV1‐3 and VVC, which in turn project to the medial parahippocampal cortex PHA1‐3 where the parahippocampal scene area (PSA) is located (Sulpizio et al. 2020; Rolls, Feng, and Zhang 2024), which in turn connect to the hippocampus (Huang et al. 2021; Ma et al. 2022; Rolls et al. 2022b; Rolls et al. 2023a; Rolls, Deco, Zhang, and Feng, 2023; Rolls 2024b; Rolls, Yan, et al. 2024).

**FIGURE 4:**
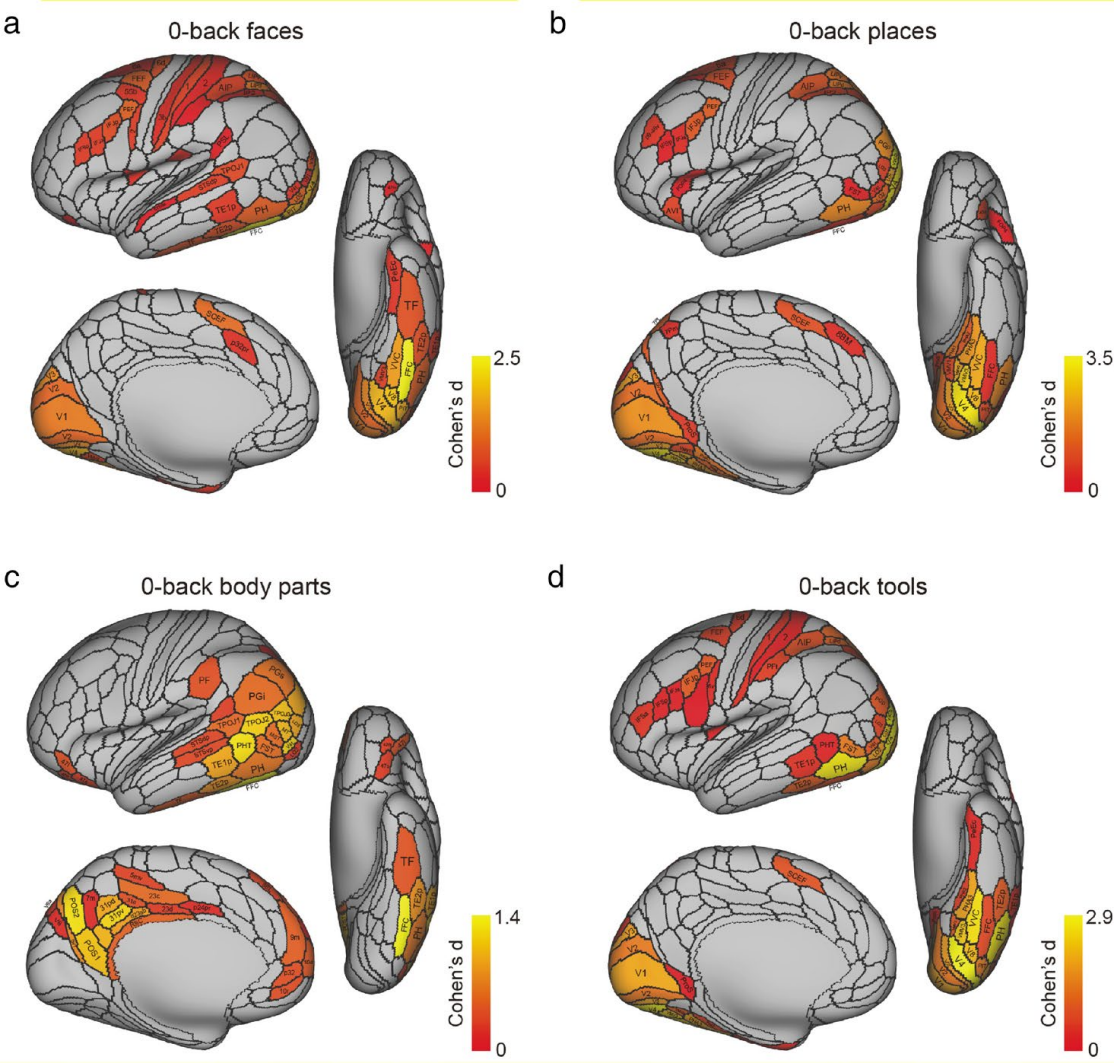
The cortical regions exhibiting significant differences in the average BOLD signal between the baseline prestimulus period preceding the zero‐back blocks (shown in Figure 1 of the original paper) before the BOLD signal had responded to the stimuli, and the last 20 timepoints within the zero‐back blocks (when the BOLD signal response to the visual stimuli was occurring) for each of the four stimulus types (faces, places, body parts, and tools) after Bonferroni correction (*α* = 0.05) across 956 participants. The effect size as indicated by Cohen's *d* is indicated. The activations are shown in red to yellow. The top 50 cortical regions with significant increases in the BOLD signal are shown out of the 180 cortical regions in the left hemisphere. The baseline prestimulus period was for the last 5 s of the 15 s fixation time and the initial 15 timepoints with a TR of 0.72 s starting when the cue was shown in a run (after Rolls, Feng, and Zhang 2024).

These connectivity analyses have been extended to show how “What” information about faces and objects reaches the human hippocampus by the ventrolateral visual cortical stream (Rolls 2023a; Rolls et al. 2023a; Rolls, Deco, Zhang, and Feng, 2023; Rolls 2024b); and how reward /goal information reaches the hippocampus from the orbitofrontal cortex (Rolls 2022; Rolls et al. 2022c). These connectivity investigations have been complemented by fMRI activation studies that add function to these pathways. It has been shown in 956 HCP participants that the medial parahippocampal PHA1‐3 and ventromedial visual VMV1‐3 are activated when viewing scenes; and that the fusiform face cortex FFC and lateral parahippocampal region TF are activated when viewing faces (Figure 4) (Rolls, Feng, and Zhang 2024). Further, it has been shown that these regions become selectively activated when performing episodic memory tasks involving object—viewed location memory or reward—viewed location memory (Rolls, Zhang, et al. 2024).

What is quite remarkable is this discovery that a “Where” representation for locations in viewed spatial scenes is encoded by a ventromedial visual cortical stream, not by the dorsal cortical visual stream (Rolls 2023a; Rolls et al. 2023a; Rolls 2024b; Rolls, Yan, et al. 2024). The proposal is that computations of the type typical of ventral visual stream cortical processing (Rolls 2012; Rolls 2021b, 2023c) are used to form feature combinations of the visual features present in different parts of scenes, using several stages of cortical processing to represent scenes based on the features found in scenes, a proposal that has been developed further as shown in Section 3.10. This is different to anything proposed for rodents.

### 3.10 How Spatial View Cells Are Built by Self‐Organizing Learning to Represent Locations in Scenes

**FIGURE 5:**
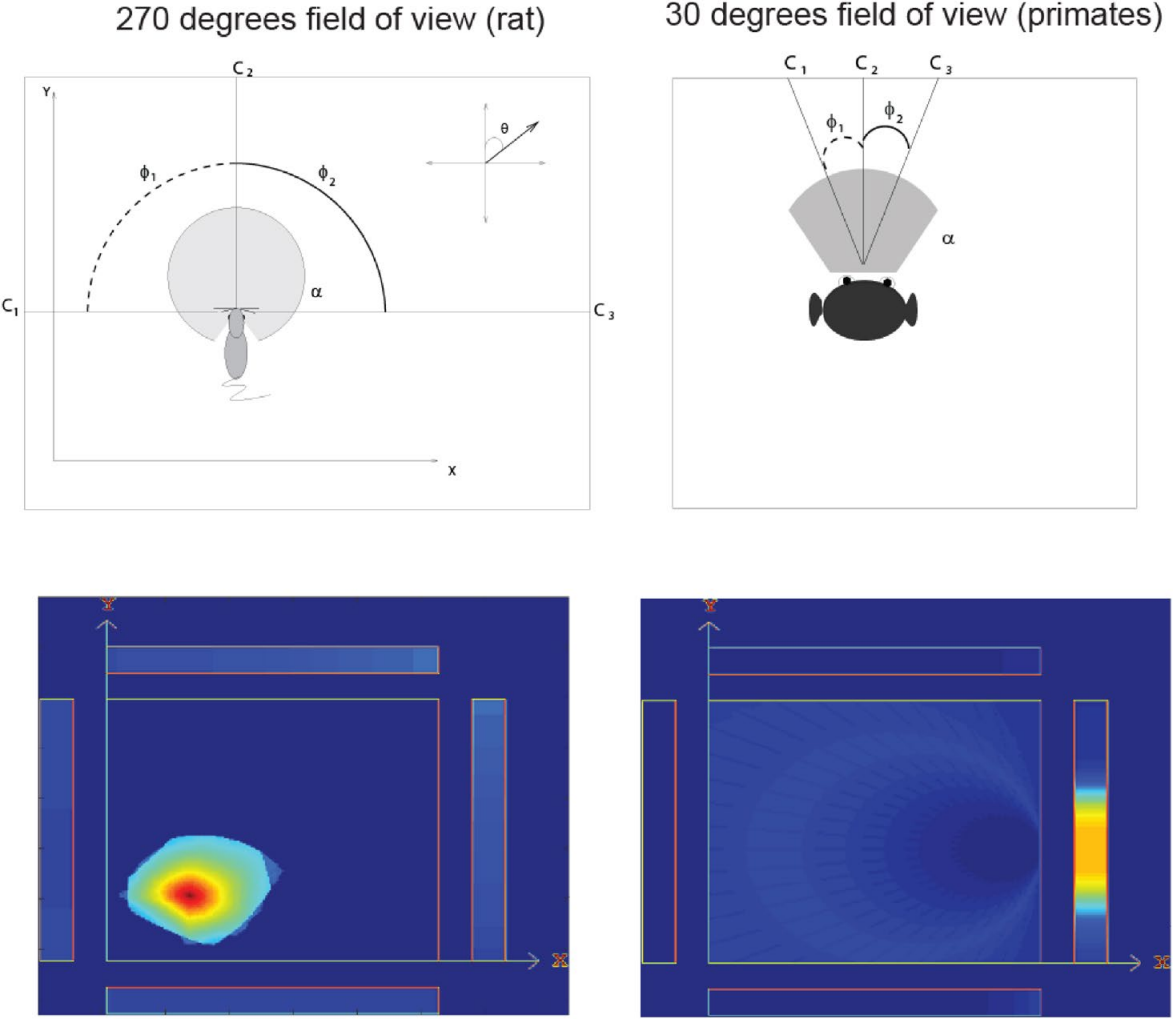
Simulation of rodent place cells (left) versus primate spatial view cells (right). The agent moved through a grid of all 200 × 200 places *x*,*y*. At each place, the head direction θ was rotated by 5° increments. Hippocampal cells are activated by a set of 3 or more landmark visual cues within the field of view of the agent *α*. The firing rates of the hippocampal neurons depended on the angles φ subtended by the landmarks. The top left shows that for a rodent with a 270° field of view a combination of such cues defines a place. The top right shows that for a primate with a 30° field of view the combination of cues defines a spatial view. The sizes of the fields of view are shown by shading. The bottom left shows that in the simulations place fields arise with a 270° field of view, and the bottom right that spatial view fields arise on one of the walls indicated by the rectangles when the field of view is 30°. High firing rates are indicated by yellow‐red. (Details are provided in De Araujo, Rolls, and Stringer (2001)).

I have taken on another key issue in understanding the primate including human hippocampal system for memory and navigation, by considering how spatial view cells, and scene representations, are built computationally in the cerebral cortex. A first approach to understanding the computations involved in forming primate spatial view neurons rather than rodent place cells is illustrated in Figure 5 (De Araujo, Rolls, and Stringer 2001). The concept builds on the fact that rodents have no fovea and a field of view in the order of 270°, whereas primates have a fovea that provides high spatial resolution for a small part of the visual field, and a highly developed cortical visual system that results in representations of objects and faces that subtend a few degrees at the retina in complex natural scenes (Rolls, Aggelopoulos, and Zheng 2003). If rodents form feature combinations of widely spaced landmarks in their 270° visual field, a place would be represented (Figure 5 left). If primates form feature combinations of features close to where they are fixating with their fovea in a visual scene, then the feature combinations formed by the neurons would represent a location in a scene, providing a model for spatial view cells (Figure 5 right). It was shown that self‐organizing learning would form these types of place or spatial view representations, depending on how wide the field of view was (Figure 5) (De Araujo, Rolls, and Stringer 2001).

**FIGURE 6:**
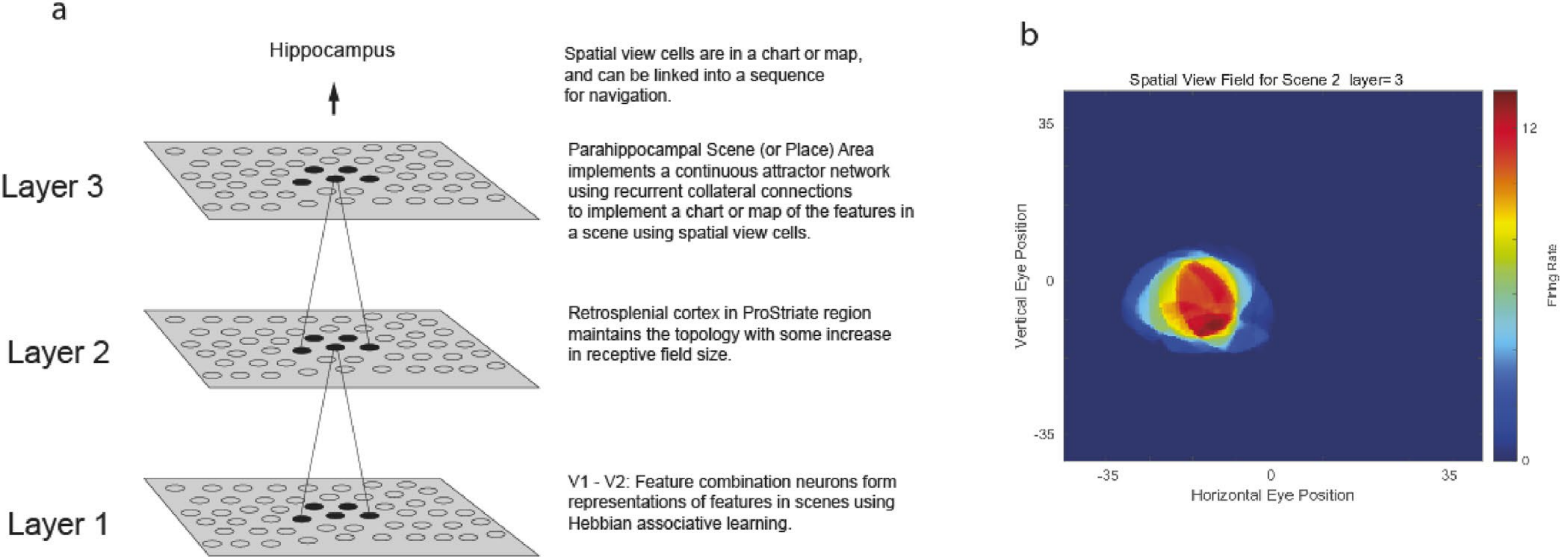
A model of a ventromedial cortical visual stream for building spatial view cells by using feature combinations, VisSceneNet. (a) The architecture of the feedforward self‐organizing network. There is a series of layers with competitive learning at each stage to build feature combination neurons for the visual features in a visual fixation scene patch, but limited convergence from stage to stage in order to maintain topography in the representations. Layer 3 uses recurrent collateral connections between the neurons to build a continuous attractor network so that the distance between the features can be represented in both the horizontal and vertical dimensions. Layer 3 may also map visual fixation scene patches to representations of whole scenes. (b) A spatial view cell built by Layer 3 of the network that responds only when a particular part of a visual scene on which the network has been trained is being viewed. The horizontal and vertical eye positions are shown in degrees (after Rolls 2024a).

A second approach is illustrated in Figure 6, which shows a model of the ventromedial cortical visual stream for building spatial view cells by using feature combinations, VisSceneNet (Rolls 2024a). Figure 6a shows the architecture of the feedforward self‐organizing network. There is a series of layers with competitive learning at each stage like VisNet (Rolls 2012; Rolls 2021b, 2023c), but unlike VisNet with only limited convergence from stage to stage in order to maintain spatial topography in the representations. Layer 3 uses recurrent collateral connections between the neurons to build a continuous attractor network so that the distance between the features can be represented in both the horizontal and vertical dimensions. In Layer 3, gain modulation by gaze direction to map visual fixation scene patches to the correct part of the whole scene representation is included. This process may be helped by inputs from the dorsal visual system to the parahippocampal gyrus that take into account gaze direction (the sum of eye and head direction) to reflect the distances between different locations in a scene, and consistent with this, some hippocampal system neurons in primates are influenced by eye movements (Ringo et al. 1994; Sobotka, Nowicka, and Ringo 1997; Sobotka and Ringo 1997; Nowicka and Ringo 2000; Buffalo 2024). Further, gaze direction may be represented in the primate hippocampus (Dun Mao, personal communication, 2024). Figure 6b illustrates a spatial view cell built by Layer 3 of the network that responds only when a particular part of a visual scene on which the network has been trained is being viewed (Rolls 2024a).

What is revolutionary about these concepts is that feature combinations are used in a type of processing typical of ventral visual cortical pathways to build “Where” representations of scenes in a ventromedial visual cortical pathway in humans and other primates. The model shows how the cortical regions such as the retrosplenial scene area and the parahippocampal scene (or place) area can build scene representations in these regions (Epstein and Kanwisher 1998; Epstein 2005; Epstein 2008; Epstein and Julian 2013; Kamps et al. 2016; Epstein and Baker 2019; Sulpizio et al. 2020; Natu et al. 2021; Rolls, Feng, and Zhang 2024) that can represent locations in scenes using spatial view cells.

### 3.11 Implications of the Discovery of Spatial View Cells for Understanding Memory and Navigation in Primates Including Humans

The discovery of spatial view cells in primates (Cahusac, Miyashita, and Rolls 1989; Rolls, Miyashita, et al. 1989; Feigenbaum and Rolls 1991; Rolls and O'Mara 1995; Rolls, Robertson, and Georges‐François 1997; Robertson, Rolls, and Georges‐François 1998; Rolls et al. 1998; Georges‐François, Rolls, and Robertson 1999; Rolls and Xiang 2005; Rolls, Xiang, and Franco 2005; Rolls and Xiang 2006) is revolutionizing our understanding of hippocampal functioning in primates including humans (Rolls 2023b; Rolls 2023a; Rolls 2023c; Rolls and Treves 2024).

First, human episodic memory typically involves remembering where people, objects and rewards or goals are in viewed scenes on particular occasions. We have shown that hippocampal spatial view cells are involved in the rapid association learning and then recall required for this type of episodic memory for objects and their locations in viewed scenes (Rolls, Xiang, and Franco 2005; Rolls and Xiang 2006), and rewards and their locations in viewed scenes (Rolls and Xiang 2005). In contrast, in rodents the focus has been on the representation of the place in which the rodent is located, and on path integration between places for navigation. The rodent findings are consistent with their much poorer visual systems, no fovea, and very wide field of view, and greater reliance on local cues such as touching and exploring local cues with the vibrissae, and olfaction. For primates, spatial view cells are naturally suited to the representations of locations being viewed, and the importance of vision in representing the identity of objects, people, and rewards such as fruit or food, so that the “What,” the “Where,” and the reward value can be associated together where they are combined, in the single attractor network in hippocampal CA3. This provides enormous adaptive value in finding rewards, people, and objects in the future, by recalling particular memories of where they have been seen. The ability of the primate hippocampus to associate viewed locations with reward value (Rolls and Xiang 2005) also has great importance in enabling the emotional or affective value to become a key part of a typical human episodic memory, and in navigation toward goals in locations “out there.”

Second, the focus in rodents of research on the hippocampal system has been on the places where the individual is located as represented by place cells, and on navigation between those places using path integration that takes into account the head direction and distance moved (O'Keefe and Dostrovsky 1971; O'Keefe 1979; Morris et al. 1982; McNaughton, Barnes, and O'Keefe 1983; Burgess and O'Keefe 1996; McNaughton et al. 1996; Samsonovich and McNaughton 1997; McNaughton et al. 2006; Moser, Moser, and Roudi 2014; Kropff et al. 2015; Moser, Moser, and McNaughton 2017). However, the discovery of spatial view cells in primates opens up new vistas in understanding navigation in primates including humans, for now navigation can be based on vision, including of landmarks in scenes. Indeed, a model has been developed of how navigation toward a sequence of landmarks provides for one important type of human navigation or piloting, with the hippocampus providing a system well suited to provide the memory for the sequence of landmarks (Rolls 2021a). If the view details are obscured, path integration over eye position, head direction, and place is proposed to take place in the dorsal visual system (Rolls 2020), to produce a representation in world‐based coordinates suitable for updating hippocampal spatial view cells by the pathways from the dorsal visual cortical regions including PGp to the medial parahippocampal cortex (Rolls 2023a, 2024b; Rolls 2024a). Remembering a sequence of spatial locations is a type of memory in which the hippocampus is involved (Weeden et al. 2009; Kesner and Rolls 2015), and may involve associations with time cells (MacDonald et al. 2011; Kraus et al. 2013; Eichenbaum 2014; Howard and Eichenbaum 2015; Rolls and Mills 2019). Landmarks are of course also potentially useful for navigation even when they are not approached. In contrast, navigation in rodents might be described as “blind navigation,” when it is described as involving path integration using head direction and distance traveled (see above). In fact, path integration is notoriously imperfect and likely to fail within a very few minutes and a short distance traveled, and most humans would not trust themselves to navigate from place to place with their eyes covered for more than a few meters and turns. Indeed, it is notable that much of the current research on the brain and navigation is based on what is found in rats and mice, nocturnal animals with poor vision that navigate in dark underground tunnels. In this situation, the discovery of spatial view cells does open up new vistas in understanding navigation, and finding goals, in humans and other primates, with excellent vision and the ability to utilize visual representations of space and visible landmarks (Rolls 2020, 2021a).

Third, the neural pathways to the hippocampus, and the information represented in the hippocampus, are very different in rodents compared to primates (Rolls 2023c, 2024b; Rolls and Treves 2024), though the actual computations performed by the hippocampal circuitry may be similar in primates and rodents (Rolls and Wirth 2018; Rolls 2023c; Rolls and Treves 2024). In rodents, the representations provided by place cells may utilize properties such as local texture, and local olfactory cues (Itskov, Vinnik, and Diamond 2011; Kesner, Hunsaker, and Ziegler 2011; Weeden et al. 2014), and it appears that hippocampal place cells may be updated by path integration by medial entorhinal cortex grid cells (Moser, Moser, and McNaughton 2017). In contrast, we have discovered that in primates including humans, the pathways to the hippocampus, and accordingly the information received by the hippocampus, are very different (Rolls 2024b; Rolls, Feng, and Zhang 2024; Rolls and Treves 2024), as follows.

1. In humans, there is the ventromedial visual cortical pathway to the hippocampus for spatial scenes that uses spatial view cells, as described in Sections 3.9 and 3.10.
2. In humans, there is also a ventrolateral visual cortical pathway via the fusiform face cortex and the lateral parahippocampal gyrus (region TF) for face and object, “What,” information to be brought to the hippocampal memory system (Rolls et al. 2023a; Rolls, Deco, Zhang, and Feng, 2023; Rolls 2024b; Rolls, Feng, and Zhang 2024; Rolls and Treves 2024). Neither of these pathways has anything closely comparable in rats and mice with their poor vision.
3. In humans, there are major pathways carrying reward information from the orbitofrontal cortex to the hippocampus, that involve regions such as the ventromedial prefrontal cortex, pregenual anterior cingulate cortex, and posterior cingulate cortex (Rolls et al. 2022c; Rolls et al. 2023b; Rolls, Wirth, et al. 2023). Not only is the rodent orbitofrontal cortex very much less well developed than in primates (Passingham 2021; Rolls 2023c), but it also is not directly comparable in what it computes with actions apparently represented in the rodent but not primate orbitofrontal cortex (Rolls 2023c), and most of these cortical regions on these pathways are poorly developed or absent in rodents (e.g., the posterior cingulate cortex) (Vogt 2009; Rolls 2019a, 2023c).

## 4 A Computational Theory for the Hippocampus and Related Systems

A second key set of discoveries is about the computations that may be performed by the hippocampus, and in the pathways that bring information to it together with how the information is represented, and about how information can be recalled from the hippocampus back to the neocortex (Rolls 1989a, 2023c; Rolls and Treves 2024).

### 4.1 Background to Computations Performed by the Hippocampus Including an Attractor Network in CA3

The background is that by the 1980s, we had made many discoveries about what was represented in the hippocampus and in other parts of the brain (see Sections 2 and 3, and Rolls 2023c), but I felt the need for a framework to understand how each part of the brain was performing its functions, and how many brain regions operated together to achieve a useful result. The answer I could see lay in understanding the computations being performed by the neuronal networks in each brain region. Key insights came when a colleague mentioned that the book *Parallel Models of Associative Memory* edited by Hinton and Anderson (1981) had just been published. This contained Chapters on autoassociation (later termed attractor) networks (Kohonen, Oja, and Lehtio 1981) and on competitive networks (see Rumelhart and Zipser 1985), and I read the stimulating books by Kohonen (1977, 1984). I very quickly programmed up autoassociation networks, competitive networks, and pattern association networks, and delivered a paper in 1985 to the Dahlem Conference on *The Neural and Molecular Bases of Learning*. Eric Kandel thought that this was sufficiently important to stop the separate workgroups of the conference, and brought everyone together for me to address the meeting about the potential of neural networks for understanding brain computations, including those performed by the hippocampus. I described how CA3 could be considered as an autoassociation network for episodic memory, that it could be used to store object‐location memories, and that the pathways from the hippocampus back to the neocortex are an important route for the information to be retrieved for long‐term storage in the neocortex. (The paper appeared in print (Rolls 1987), and is on my website. In the same year, another paper appeared that also proposed that the CA3 cells operate as an autoassociation network (McNaughton and Morris 1987). The important earlier work of Marr (1971) was cited by both of these papers, though Marr had not identified CA3 as an autoassociation matrix memory.)

### 4.2 Formulation of a Theory of Recall of Memories From the Hippocampus Back to the Neocortex

**FIGURE 7:**
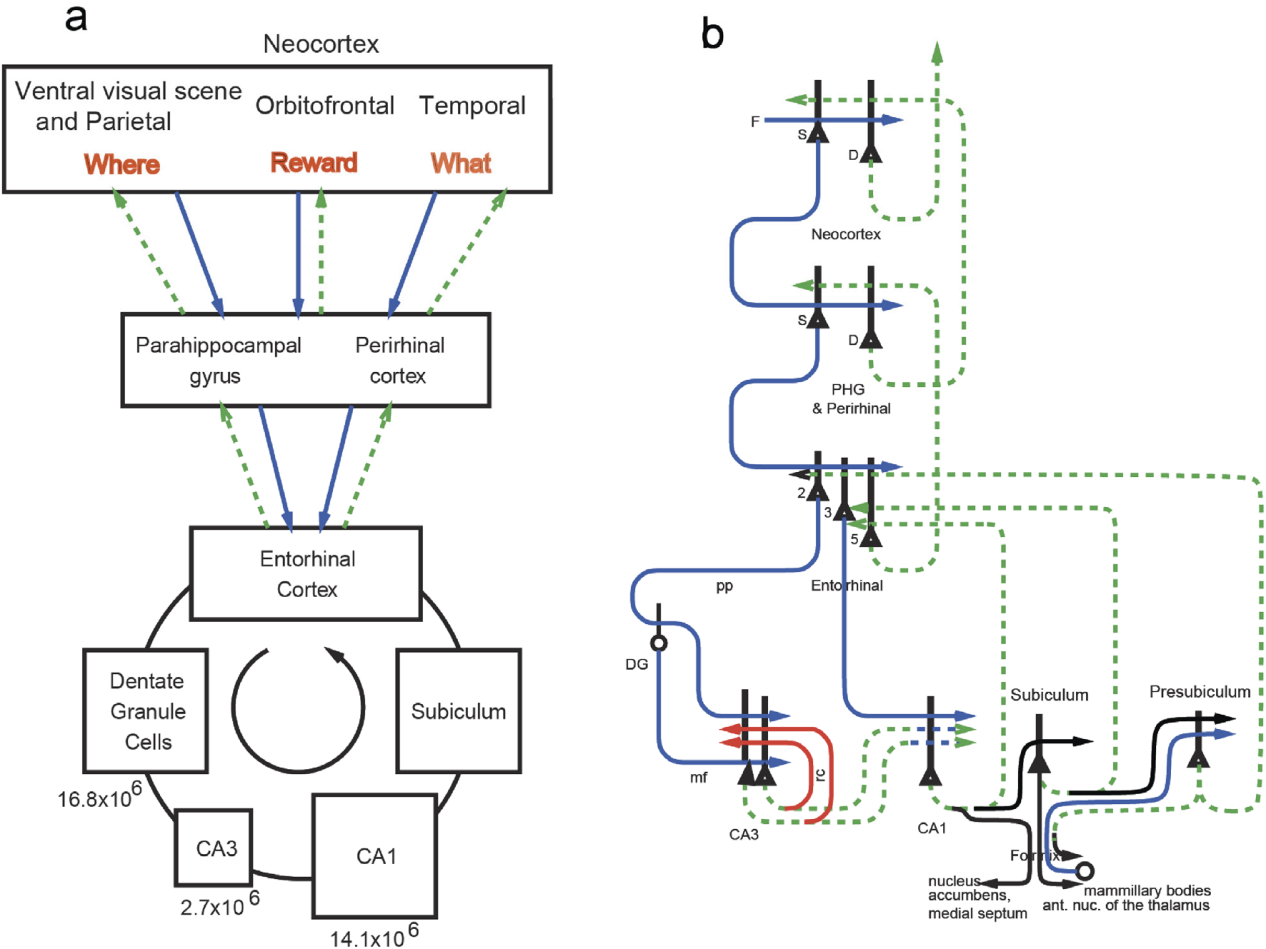
The human/primate hippocampus receives neocortical input connections (blue) not only from the “what” temporal lobe and “where” parietal and ventral visual scene areas, but also from the “reward” prefrontal cortex areas (orbitofrontal cortex, vmPFC, and anterior cingulate cortex) for episodic memory storage; and has return backprojections (green) to the same neocortical areas for memory recall. There is great convergence via the parahippocampal gyrus, perirhinal cortex, and dentate gyrus in the forward connections down to the single network implemented in the CA3 pyramidal cells, which have a highly developed recurrent collateral system (red) to implement an attractor episodic memory by associating the what, where and reward components of an episodic memory. (a) Block diagram. (b) Some of the principal excitatory neurons and their connections in the pathways. Time and temporal order are also important in episodic memory, and may be computed in the entorhinal‐hippocampal circuitry (Rolls and Mills 2019). D: Deep pyramidal cells. DG: Dentate Granule cells. F: Forward inputs to areas of the association cortex from preceding cortical areas in the hierarchy. mf: mossy fibers. PHG: parahippocampal gyrus and perirhinal cortex. pp: perforant path. rc: recurrent collateral of the CA3 hippocampal pyramidal cells. S: Superficial pyramidal cells. 2: pyramidal cells in layer 2 of the entorhinal cortex. 3: pyramidal cells in layer 3 of the entorhinal cortex. The thick lines above the cell bodies represent the dendrites. The numbers of neurons in different parts of the hippocampal trisynaptic circuit in humans (Rogers Flattery et al. 2020) are shown in (a), and indicate very many dentate granule cells, consistent with expansion encoding and the production of sparse uncorrelated representations prior to CA3 (Rolls 2016d, 2021c).

My next key step in the development of the theory of the hippocampus was to produce a theory of how information could be recalled from the hippocampus back to the neocortex. Marr (1971) had promised a theory for this, but never produced a theory. My theory is that the backprojection pathways from the hippocampus to the neocortex (shown in green in Figure 7) are active during the learning of the episodic memory, and form associatively modifiable synapses onto the neocortical neurons that are active during the learning (Rolls 1989d; Rolls 1989b; Rolls 1989c; Rolls 1989a). Each stage of the backprojection pathways is thus formally a pattern association network. (Descriptions of autoassociation/attractor, pattern association, and competitive networks, and Matlab and Python programs to illustrate their operation, are provided by Rolls (2023c).) Later, during recall, the neocortex provides a partial recall cue (such as the object, then the system needs to recall the location of the object), completion of the whole “What”—“Where” memory occurs in the CA3 autoassociation/attractor network, and then the correct “Where” neurons are recalled by the multiple stage backprojection pattern association pathways back to the neocortex that are illustrated in green in Figure 7 (Rolls 1989a). This theory of recall from the hippocampus to the neocortex (Rolls 1989a) (see also Treves and Rolls 1994; Rolls and Treves 2024; Rolls, Zhang, and Feng 2024) was adopted later by McClelland, McNaughton, and O'Reilly (1995), though they pointed out that the pattern association process for the backprojections might not need to be learned simultaneously during the storage at every stage as long as it occurred at one or more stages during the storage, if the other stages had already been set up to implement the same pattern association process for the backprojection synapses (see Rolls and Treves 2024).

### 4.3 The Development of Quantitative Approaches to Understanding the Storage and Recall of Information in Hippocampal and Other Cortical Networks With Sparse Distributed Coding, Graded Firing Rates, and Asymmetric Diluted Connectivity

At about this time (1990), the theoretical physicist Daniel Amit, who wrote an important book on attractor networks (Amit 1989), visited Oxford and gave a research seminar on attractor networks. In discussion after the seminar, I pointed out to him that neurons in the brain have sparse distributed representations (with a sparseness often close to 0.1), graded firing rates (typically in the range 0–100 spikes/s), and diluted connectivity (typically 0.04–0.1), whereas the attractor networks analyzed by theoretical physicists generally used binary neurons with firing rates of for example either +1 or −1, full connectivity guaranteeing symmetric connections between neurons with use of an associative learning rule and hence a stable basin of attraction, and a sparseness of 0.5 (i.e., 50% of the neurons active for any one vector of firing rates to be stored in the network) (Hopfield 1982; Amit 1989; Hertz, Krogh, and Palmer 1991). I emphasized that it would be very important if the physics‐based approaches to attractor networks could be extended to networks with sparse representations, diluted connectivity, and graded firing rates, in order to produce quantitative estimates of the storage capacity, stability, and so forth of biologically realistic attractor networks. I give great credit to Daniel Amit for somehow arranging for his wonderful student the theoretical physicist Alessandro Treves to visit my lab at Oxford for several years as a postdoctoral scientist and to develop the theory of the hippocampus further to become fully quantitative, when we extended the theory of attractor networks and similar networks to deal with the biologically realistic case of asymmetric diluted connectivity, sparse representations, and graded firing rates (Rolls and Treves 1990; Treves and Rolls 1991; Rolls and Treves 1998). These were key advances that provide a quantitative foundation for the analysis of biologically plausible network in the brain, as described next.

We started with pattern association networks, used for association learning in the backprojection pathways from the hippocampus, and in other brain regions such as the orbitofrontal cortex (Rolls 2023c). We were able to show that the maximum number of patterns, *p*_max_, that can be stored and correctly retrieved with sparse representations, and graded firing rates, in a pattern association network is approximately

(1) $$p_{\max}=\frac{C}{\left[a_{o}\log\left(\frac{1}{a_{o}}\right)\right]}$$

where *C* is the number of synaptic connections onto each neuron, and *a*_o_ is the sparseness of the output representation (Rolls and Treves 1990). The sparseness *a* in Equation (1) would be for binary neurons the proportion of the population of neurons with high firing rates, and has been generalized to the case with graded firing rate neurons, which are found in the brain (Rolls and Tovee 1995; Franco et al. 2007; Rolls and Treves 2011; Rolls 2023c), to

(2) $$a={\left(\sum_{i=1,N}r_{i}/N\right)}^{2}/\sum_{i=1,N}\left({r_{i}}^{2}/N\right)$$

where *r*_*i*_ is the firing rate (e.g., spikes/s, typically in the range 0–100 spikes/s) of the *i*'th neuron in the set of *N* neurons (Rolls and Treves 1990; Treves and Rolls 1991). The sparseness ranges from 1/*N*, when only one of the neurons responds to a particular stimulus (a local or grandmother cell representation; Rolls and Treves 2011), to a value of 1.0, attained when all the neurons are responding at the same rate to a given stimulus (Treves and Rolls 1991; Franco et al. 2007; Rolls and Treves 2011; Rolls 2023c).

Next, the storage capacity of attractor (autoassociation) networks with asymmetric diluted connectivity, sparse representations, and graded firing rates was investigated, and it was shown that the memory capacity of an attractor network in terms of *p* the maximum number of patterns that can be stored and correctly retrieved is approximately

(3) $$p=\frac{\mathrm{Ck}}{\left[a\;\log\left(\frac{1}{a}\right)\right]}$$

where *C* is the number of recurrent collateral synaptic connections onto each neuron, *a* is the sparseness of the representation, and *k* is a factor that depends weakly on the detailed structure of the firing rate distribution, on the connectivity, and so forth, but is roughly in the order of 0.2–0.3 (Treves 1991a, 1991b; Treves and Rolls 1991; Rolls 2023c). For *C* = 12,000 synapses per neuron as in the rat CA3, Equation (3) evaluates to approximately 12,000 memory patterns when the sparseness *a* is close to 0.1. This research advances the Hopfield model (Hopfield 1982) by incorporating sparse representations, diluted connectivity, and graded firing rates, and does not rely on the binary synaptic weights considered by Marr (1971), which is often referred to as the Willshaw model (Willshaw, Buneman, and Longuet‐Higgins 1969; Rolls and Treves 1998). An advantage of neurons with graded firing rates is that this prevents or limits the chaotic attractor states that can arise with binary neurons and with asymmetric dilution of the connectivity, and this is a point that is still insufficiently appreciated. Further details of these and related analyses are available (Treves 1990, 1991a, 1991b; Rolls and Treves 1998, 2024).

### 4.4 The Development of a Quantitative Theory of the Operation of Hippocampal CA3 as an Attractor Network for Memory Storage and Recall

**FIGURE 8:**
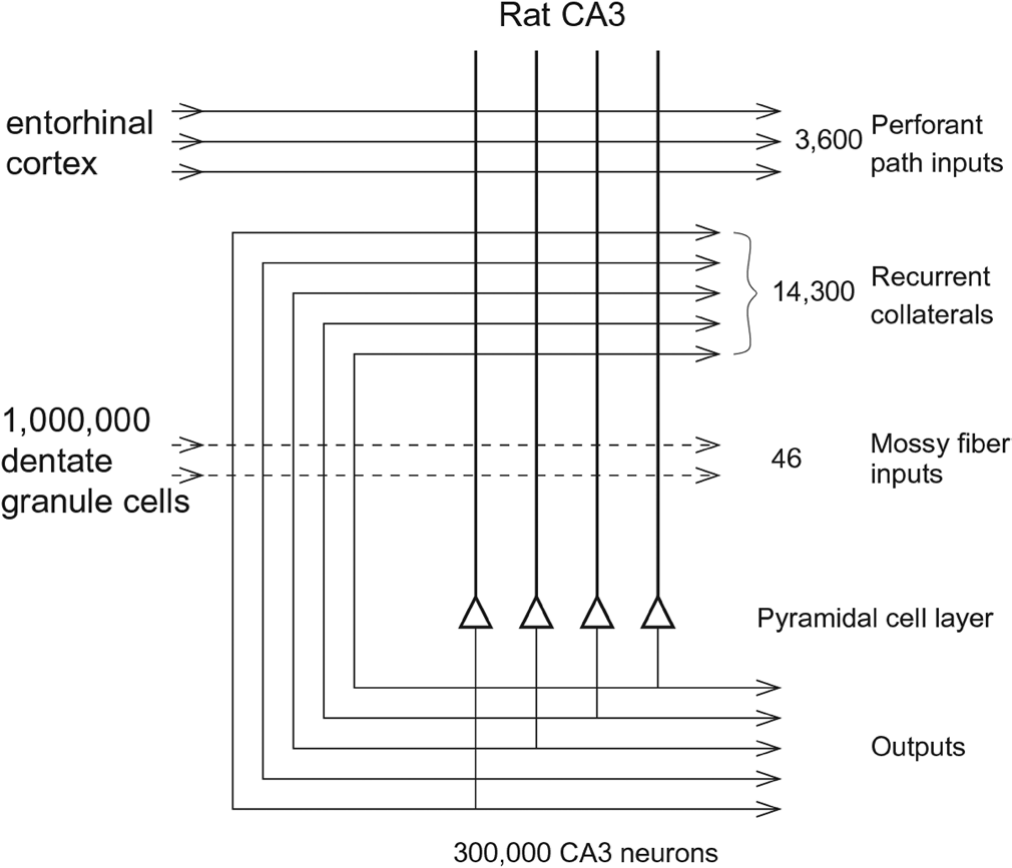
The numbers of connections from three different sources onto each CA3 cell from three different sources in the rat (after Amaral, Ishizuka, and Claiborne 1990; Treves and Rolls 1992; Rolls and Treves 1998). The number of recurrent collaterals per CA3 neuron is updated with data from Watson et al. (2024). The number of perforant path inputs from the entorhinal cortex may be a limiting factor in the number of memories that can be recalled from the hippocampal system, and counts of these numbers in humans will be of great interest. The numbers shown are for one hemisphere.

The results shown in Equations (1) and (3) provide key foundations of the analysis included in the quantitative theory of hippocampal function that we developed next (Treves and Rolls 1992; Rolls and Treves 1994; Treves and Rolls 1994; Rolls and Treves 2024). To help develop the hippocampal theory quantitatively, I visited David Amaral's lab, and asked him what estimates he might be able to provide for the numbers of synaptic connections per neuron, and the numbers of neurons, in different parts of the hippocampal circuit. David Amaral was very helpful, and this resulted in the values shown in Figure 8, which were published (Amaral, Ishizuka, and Claiborne 1990).

The quantitative theory of CA3 and its inputs from the dentate gyrus and entorhinal cortex included the following key concepts (Treves and Rolls 1992).

#### 4.4.1 The Storage Capacity of CA3

The storage capacity of CA3 is set by the number of connections *C* per neuron for the recurrent collaterals, not by the number of neurons *N*, and given a sparseness of about 0.1, is about 12,000 memories in the rat, given that the number of recurrent collateral connections per CA3 neuron is approximately 12,000 in the rat (Figure 8, though now updated to 14,300 in the rat with new data from Watson et al. (2024)). It would be very useful indeed to know this number *C* in humans, for that would help to provide an estimate of the number of memories that can be stored in the human hippocampus. The update is to *C* = 17,500 in humans (Watson et al. 2024). This is important, for beyond that limit, an inability sets in to retrieve memories from an attractor network, which needs therefore to forget by for example gradually over‐writing previous memories with each new random set of CA3 neurons allocated to a new memory, and this is very relevant to understanding the gradient of retrograde amnesia, and why some forgetting is so important (Rolls 2023c; Rolls and Treves 2024) (see also Kim and Fanselow 1992; McClelland, McNaughton, and O'Reilly 1995).

#### 4.4.2 The Dentate Granule Cell/Mossy Fiber Connectivity to CA3

The dentate granule cell/mossy fiber connectivity to CA selects a new random subset of CA3 neurons for each new memory to be stored in the hippocampus. Part of the computational utility of this is that this minimizes the correlations between the sets of CA3 neurons that are active for each memory pattern to be stored, which is important in order not to diminish the memory capacity of autoassociation/attractor networks (Rolls 2023c). Part of what I have termed this pattern separation (Rolls 1989a) is achieved by the low contact probability of the mossy fibers with CA3 neurons, which is 46 synapses per CA3 neuron/10^6^ dentate granule cells = 0.000046 (Treves and Rolls 1992). (This can now be updated to 220 mossy fiber inputs per CA3 neuron in humans from 15 × 10^6^ dentate granule cells (Watson et al. 2024), for which the same arguments apply.) Other contributions to this pattern separation include the competitive learning performed by the dentate granule cells, the very sparse representation in the dentate granule cells which can be considered to perform expansion recoding, and the sparse coding in CA3 (Rolls 1989a, 2013, 2016d, 2023c; Rolls and Treves 2024) (see also McNaughton, Chen, and Markus 1991; Hasselmo and Wyble 1997). This random new set of CA3 neurons selected for each new memory is what in the caching chickadee was termed a bar code for each new cache or memory (Chettih et al. 2024). This randomizing process is likely to be facilitated by neurogenesis of dentate granule cells (Rolls 2016d; Gage 2024).

#### 4.4.3 The Entorhinal Cortex to CA3 Synapses Are Used to Retrieve the Memory in CA3

We showed that the dentate mossy fiber to CA3 synapses are too few (46 synapses per CA3 neuron in the rat, Figure 8) to recall more than a few memories (Treves and Rolls 1992). We further showed that the entorhinal cortex to CA3 synapses if associatively modified at the time of memory storage could later be used to recall the random set of CA3 neurons that had been set up for a particular memory (Treves and Rolls 1992). The memory capacity as shown in Equation (1) for a pattern association network would be moderately high, approximately 3600 memories given the 3600 entorhinal to CA3 synapses per CA3 neuron and a sparseness of the CA3 neuron representation in the region of 0.1. However, the number of entorhinal to CA3 synapses if really as low as 3600 in rodents would not enable the higher memory capacity of CA3 as an attractor network with 12,000 synapses per neuron to be fully realized (Rolls, Zhang, and Feng 2024), and further estimates especially in humans of the number of entorhinal synapses on to each CA3 neuron would be of great interest.

### 4.5 A Quantitative Theory of the Recall of Memories From the Hippocampus to the Neocortex

The theory I developed (Rolls 1989a) was that recall from the hippocampus to the neocortex was implemented after the recall of the whole set of neurons in CA3 for a memory by (1) competitive learning in CA1 to make an efficient recall cue in which the parts of a memory, necessarily separate in CA3 for the autoassociation, could be combined to make a more efficient memory recall cue; and (2) the CA1 acting on a series of cortical stages back to the neocortex involving pattern association learning for the backprojections shown in green in Figure 7. The multistage pathway for the backprojections from CA1 to the neocortex was understood to provide a way stage by stage of increasing the numbers of neurons that could be activated during recall, in order to reach the vast number of neurons in the neocortex (Rolls 1989a, 2023c). If CA1 were to connect directly to the final neocortex regions, each CA3 neuron would have to send in the order of 10,000 synapses to each of hundreds of millions of neocortex neurons, which is biologically implausible, hence the need for a multistage backprojection pathway from the hippocampus to the neocortex (Rolls 1989a) (Figure 7).

But the next question that it was important to address was: what is the memory recall capacity of a series of pattern association networks used in the backprojection pathways (Figure 7) (Treves and Rolls 1994)? How many memories could be recalled from the hippocampus to the neocortex? This held Alessandro Treves and I up a little, until one evening just before Christmas the idea emerged in discussions that a multistage series of pattern association networks could be analyzed with the quantitative methods used to analyze the capacity of an attractor network, for each backprojection stage could be considered as one iteration round an autoassociation (attractor) network (Treves and Rolls 1994). The result was that the memory recall capacity *p* of the multistage backprojection system could now be expressed as:

(4) $$p=\frac{\mathrm{Ck}}{\left[a\;\log\left(\frac{1}{a}\right)\right]}$$

where *C* is now the number of backprojection synapses onto any one neuron in the backprojection pathway, *a* is the sparseness of the representation in now the backprojection pathway (green in Figure 7), and *k* is a factor that depends weakly on the detailed structure of the firing rate distribution, on the connectivity, and so forth, but is roughly in the order of 0.2–0.3. (I remark in this context that for interdisciplinary research to flourish, I have found it very useful for scientists to work and discuss together in the same place for some considerable time, for then understanding develops that can lead to new insights when their different expertise can be combined.)

The implication is that there should be many backprojection synapses on each neuron for the pathway back to the neocortex shown in green in Figure 7. To enable the same number of memories to be recalled to neocortex as could be stored in CA3, with its 12,000 synapses per neuron for the recurrent collaterals (Figure 8), the number of backprojection synapses on each neuron in the cortical backprojections should be close to 12,000. In fact, the number of backprojection synapses onto each cortical neuron is high, with estimates in the order of 10,000 per neuron (Rolls 2023c).

This theory (Treves and Rolls 1994) remains the only quantitative theory of the recall of memories from the hippocampus to the neocortex (Rolls and Treves 2024). The hippocampus can be seen as providing a pointer, or in fact pointers (Rolls, Zhang, and Feng 2024), back to the neocortex, and overall sets up an arbitrary set of CA3 neurons for each memory, and then uses the backprojections as pointers back to the neocortex to recall the neocortical neurons in different cortical regions that were active during storage back into activity during recall (Rolls 2023c). The concept that the hippocampus might operate as a pointer system reminds one of the pointer or indexing functionality discussed previously but with no theory and mechanism for how anything like that could work (Teyler and DiScenna 1986).

This theory provides a quantitative account of why there are as many backprojections between each pair of cortical regions as there are forward connections, and is the only quantitative theory for this fundamental property of neocortex architecture (Rolls 2016e, 2023c). These backprojections are used not only for memory recall, but also for top‐down attention, another key feature of neocortical design that is considered in my research (Rolls 2016e, 2023c).

### 4.6 Is the CA3 Network a Memory System or an Idiothetic Update System?

**FIGURE 9:**
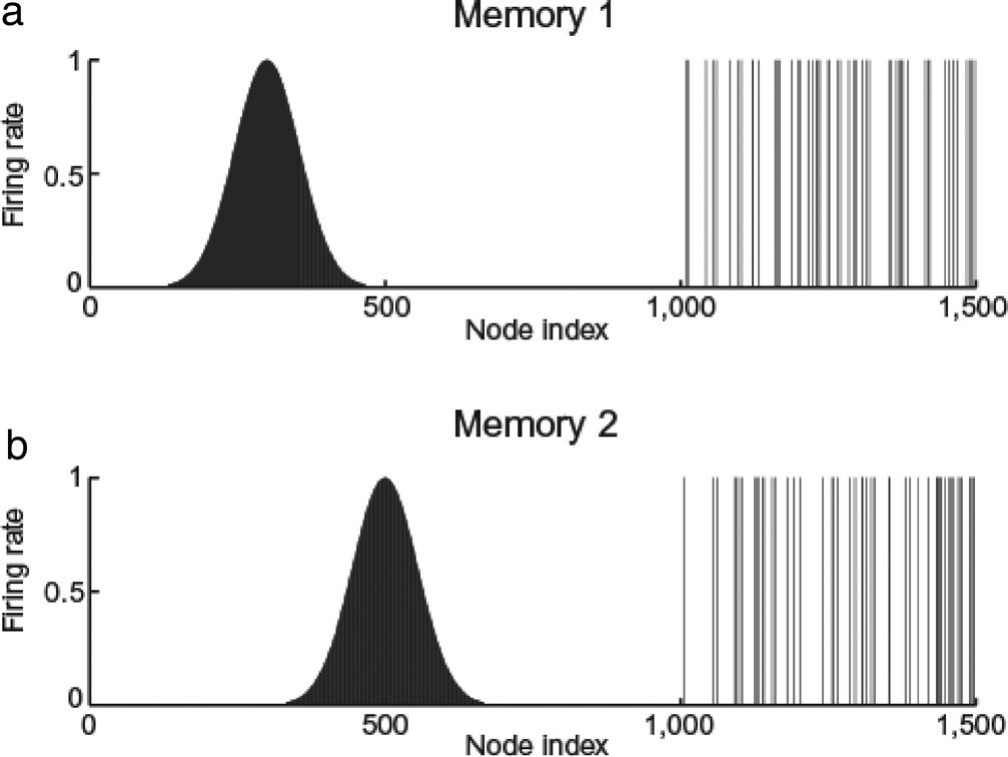
The types of firing patterns stored in continuous attractor networks are illustrated for the patterns present on neurons 1–1000 for Memory 1 (when the firing is that produced when the spatial state represented is that for location 300), and for Memory 2 (when the firing is that produced when the spatial state represented is that for location 500). The continuous nature of the spatial representation results from the fact that each neuron has a Gaussian firing rate that peaks at its optimal location. This particular mixed network also contains discrete representations that consist of discrete subsets of active binary firing rate neurons in the range 1001–1500. The firing of these latter neurons can be thought of as representing the discrete events that occur at the location. Continuous attractor networks by definition contain only continuous representations, but this particular network can store mixed continuous and discrete representations, and is illustrated to show the difference of the firing patterns normally stored in separate continuous attractor and discrete attractor networks. For this particular mixed network, during learning, Memory 1 is stored in the synaptic weights, then Memory 2, and so forth, and each memory contains part that is continuously distributed to represent physical space, and part that represents a discrete event or object. The spatial and object representations are bound together by being simultaneously present when the event is stored. (From Rolls, Stringer, and Trappenberg 2002, where further details can be found).

Some previous approaches have suggested that the hippocampal CA3 recurrent network might operate as a path integration system for self‐motion update from place to place (McNaughton et al. 1996; Samsonovich and McNaughton 1997). One problem with this is that if CA3 is also used for episodic memories, to associate locations using a continuous spatial representation, and objects using a discrete representation with a random set of neurons firing for each object (Rolls, Stringer, and Trappenberg 2002) (Figure 9), then the energy landscape of the network would be too “bumpy” to support a continuous attractor which utilizes a flat energy landscape to enable movement freely and continuously from location to location (Rolls 2023c; Rolls and Treves 2024). But in any case, it appears that path integration in rodents may be performed before the hippocampus, in the medial entorhinal cortex, by grid cells (Giocomo, Moser, and Moser 2011; Moser, Moser, and Roudi 2014; Moser, Moser, and McNaughton 2017). And in primates including humans in which eye movements are required to be part of the path integration system, it is likely that path integration occurs in the dorsal visual system, and is then communicated to the medial parahippocampal cortex via the intraparietal visual cortical regions and PGp (Rolls 2020, 2023c; Rolls 2023a; Rolls 2024b; Rolls and Treves 2024).

Given these points, it is proposed that the hippocampal CA3 system may be much more involved in episodic memory than in the path integration that can be useful in the dark for navigation (Rolls 2023c; Rolls 2023a; Rolls 2024b; Rolls and Treves 2024). The hippocampal episodic memory system could of course be useful in this scenario for navigation using visual cues and landmarks, by providing a sequence memory for the locations in spatial scenes to be used for navigation (Rolls 2021a, 2023c).

### 4.7 New Developments in Understanding Hippocampal Computation

I have continued to develop further all of these and many other concepts about hippocampal function, and proposed to Alessandro Treves that 30 years after some of our original publications (Rolls and Treves 1994; Treves and Rolls 1994), we might provide an update. We worked together on this, and further new developments in the theory of hippocampal function include the following (Rolls and Treves 2024).

#### 4.7.1 “What” Inputs to the Primate Including Human Hippocampus for Episodic Memory

As summarized in Section 2.3 and in detail elsewhere (Rolls 1984; Rolls, Baylis, and Hasselmo 1987; Hasselmo, Rolls, and Baylis 1989; Hasselmo et al. 1989; Booth and Rolls 1998; Rolls 2000; Rolls 2012; Rolls 2021b, 2023c), we have discovered that there is a pathway to the macaque IT visual cortex for developing representations of objects and faces that are invariant with respect to translation, size, contrast, spatial frequency, and even in some cases to view. The invariance in these representations is ideal for an input to a memory system such as the hippocampus, for any view, and so forth of the person or object seen later will act as an appropriate input to the hippocampal memory system for retrieval of the whole memory with its “Where” and reward components.

Moreover, we have shown that the identity of faces and objects is in an excellent form with sparse distributed representations in which the information increases linearly with the number of neurons (Rolls, Treves, and Tovee 1997; Rolls et al. 1997; Rolls and Treves 2011) for an input to a memory system such as the hippocampus.

Further, we have shown that the connectivity of the “What” system in humans reaches the hippocampus via the fusiform face cortex, and lateral parahippocampal cortex region TF (Rolls et al. 2023a; Rolls 2024b; Rolls, Feng, and Zhang 2024; Rolls and Turova 2025).

Very little that is equivalent has been described in rats and mice, yet this is a key component of the episodic memory system that I have described in primates including humans (Rolls 2023c), contributing to the new understanding of hippocampal function in primates including humans.

#### 4.7.2 “Where” Inputs to the Primate Including Human Hippocampus for Episodic Memory

As summarized in Section 3 and in detail elsewhere (Rolls 2023c; Rolls 2023a; Rolls, Feng, and Zhang 2024), the “Where” input to the hippocampus is about the viewed location in a spatial scene, which is ideal for a human episodic memory system that prototypically enables storage and later recall of where we have seen objects, people, and rewards (goals), in space “out there” that is being viewed. The allocentric location in the viewed scene is again an ideal input to the hippocampal episodic memory system, for the input is invariant with respect to the eye position, head direction, facing direction, and place when the location “out there” in the scene is being viewed. Moreover, I have described with colleagues the effective and functional connectivity and tractography of a new ventromedial cortical visual stream to the human hippocampus for scene information (Rolls et al. 2023a; Rolls 2024b; Rolls, Yan, et al. 2024), shown how the cortical regions along this pathway are activated by scenes compared to faces, tools and body parts with data from 956 participants (Rolls, Feng, and Zhang 2024), and have developed a theory and model of how the scene representations may be built along this pathway (Rolls 2024a).

Very little that is equivalent has been described in rats and mice, yet these are key components of the episodic memory system that I have described in primates including humans (Rolls 2023c), contributing to the revolution in our understanding of hippocampal function in primates including humans.

#### 4.7.3 Reward/Affective Inputs to the Primate Including Human Hippocampus for Episodic Memory

The reward/affective input is a key component of episodic memory, and we have shown that this is represented in the primate hippocampus and can be combined with location information (Rolls and Xiang 2005).

We have also shown the pathway in humans by which reward information from the orbitofrontal cortex (Rolls 2014b, 2019a, 2023d) reaches the hippocampal episodic memory system in part via the ventromedial prefrontal cortex, pregenual anterior cingulate cortex, and posterior cingulate cortex (Rolls et al. 2022c; Rolls, Wirth, et al. 2023). The computation performed by the human hippocampus can thus be considered to include episodic memory storage and recall with “What,” “Where,” and reward/affective value components (Rolls 2023c) (Figure 7).

These pathways are also proposed to be important in providing the goals for navigation (Rolls 2023c).

#### 4.7.4 The Roles of Reward in Memory Consolidation

We have also shown that there is an input from the orbitofrontal cortex reward/emotion system to the basal forebrain/septal system where there are cholinergic neurons that project to the neocortex and hippocampus (Rolls et al. 2022c), and have generated the theory that this contributes to memory consolidation (Rolls 2022) by increasing long‐term potentiation (Hasselmo 1999; Hasselmo and McGaughy 2004; Hasselmo and Giocomo 2006; Hasselmo and Sarter 2011; Zaborszky et al. 2018). This theory is supported by evidence that primate basal forebrain neurons can be activated by rewarding, punishing, or novel visual stimuli (Wilson and Rolls 1990a, 1990b, 1990c; Rolls 2023c).

In addition, I have proposed that when an episodic memory is recalled to the neocortex from the hippocampus with a reward (or punishment) value component, that episodic memory may be rehearsed and thought about more because it may be of value, and that extra processing may enhance memory consolidation into semantic or long‐term memory (Rolls 2022).

#### 4.7.5 Reentrant Neocortex‐Hippocampus‐Neocortex Processing

**FIGURE 10:**
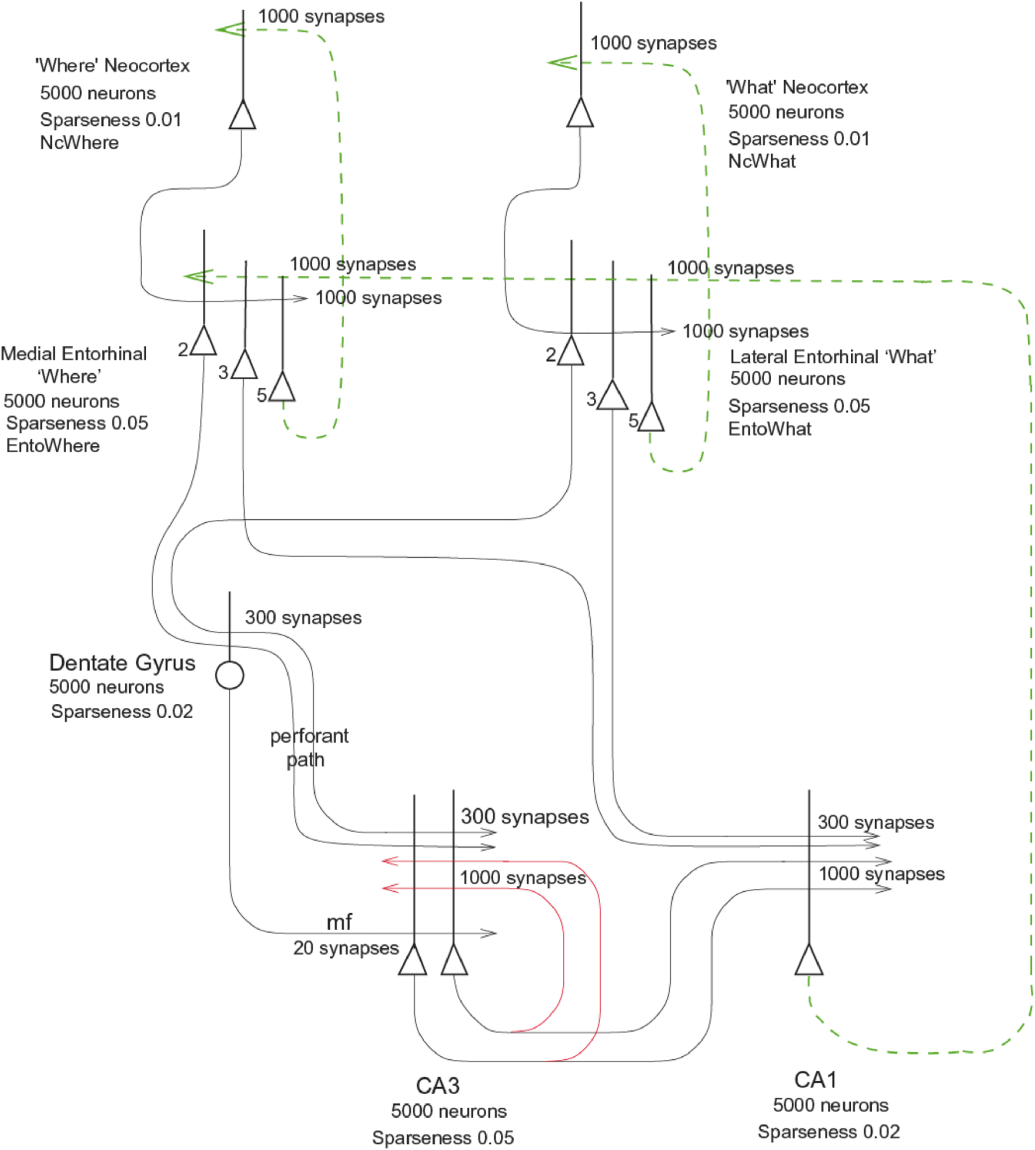
Simulation of neocortical “what” and “where” inputs to the hippocampus for the storage of episodic memory, and for the recall of “what” (object or face) and “where” (spatial view) information back to the “what” and “where” neocortex. The pyramidal cell bodies are shown as triangles, the dendrites as the thick lines above the cell bodies, and the axons as thin lines terminated with an arrow. The numbers of synapses shown are the numbers on any one neuron. The backprojection pathways for memory recall are shown in dashed green lines; and in red the CA3 recurrent collaterals via which “what” and “where” representations present at the same time can be associated during episodic memory storage, and via which completion of a whole memory from a part can occur during recall. All synapses are associatively modifiable except for the dentate gyrus (DG) mossy fiber (mf) synapses on the CA3 pyramidal cells. The dentate granule cells, the CA1 cells, and the entorhinal cortex inputs from the neocortex operate as competitive networks. The CA3 cells operate as an autoassociation attractor network to implement completion. The backprojection connections shown in green operate as pattern association networks (Rolls 2023c) (after Rolls, Zhang, and Feng 2024).

In an integrate and fire model of the dynamics of neocortical‐hippocampal‐neocortical processing, it has been found that after a partial cue has retrieved memory back to the neocortex (Figure 10), the neocortex then in this continuous time model sends input back to the hippocampus, which returns it back to the neocortex, in a continuous positive feedback loop (Rolls, Zhang, and Feng 2024). A theory is being developed that this may enable several different neocortical representations (e.g., What, Where, and Reward) to be kept active by what are effectively backprojection pointers back to specific neocortical representations in several different cortical regions. Keeping these several neocortical representations active for a short period of perhaps seconds may help the neocortex to build long‐term representations in, for example, the anterior temporal lobe semantic regions (Rolls et al. 2022a; Rolls 2023c; Rolls, Zhang, and Feng 2024).

## 5 Concluding Remarks

To understand a brain computation, a set of integrated interdisciplinary discoveries is needed, including empirical discoveries at the neuronal level about what is represented in connected brain regions, and computational neuroscience theories about how the computations are performed. That approach with a set of integrated interdisciplinary discoveries to understand the operation of brain systems is described here and in Brain Computations and Connectivity (Rolls 2023c), and contrasts with the single advance that is sometimes recognized by awards in neuroscience.

Research in nocturnal animals such as rats and mice that live in underground tunnels has made advances in understanding “blind” navigation from place to place using path integration over the direction and distance traveled, but is not useful in humans for most of the navigation that we perform. The discoveries described here emphasize the roles of vision in the functions of the hippocampus in memory and navigation in primates including humans. It is anticipated that the empirical and computational discoveries described here provide the foundation for new vistas and a revolution in the understanding of brain systems involved in memory and navigation in humans and other primates.

## Conflicts of Interest

The author declares no conflicts of interest.

## Supporting information


**SUPPORTING INFORMATION** Videos that illustrate the responses of macaque hippocampal spatial view cells are provided as Supporting Information. One example of a hippocampal spatial view cell is in file az033.mp4, which illustrates a small part of the data from this neuron that was included in the analysis of the coordinate system used by spatial view neurons (Georges‐François, Rolls, and Robertson 1999). The enclosure is the central square, the four walls are the rectangles surrounding the square with the height on the wall indicated by the distance in the wall rectangle away from the center of the diagram, and a red dot is added to this wall plot whenever the cell fires an action potential. The position and head direction of the macaque are indicated by the triangle, and the eye gaze direction by the line projected to the edge of the enclosure, which is black when the cell is not firing, and red when the cell fires. The firing of other spatial view cells during active locomotion are illustrated in Supporting Information files: av232.mp4, av191.mp4, and az110(2).mp4. It should be noted that all the statistical analyses on these cells were performed with more extensive data, and took into account occupancy, as described elsewhere (Rolls et al. 1998; Georges‐François, Rolls, and Robertson 1999).

## Data Availability

Programs written in Matlab (which also run under the freeware Octave) to illustrate the operation of autoassociation (attractor) and related networks are available at https://www.oxcns.org/NeuronalNetworkSimulationSoftware.html with a description of the software in Appendix D of *Brain Computations and Connectivity* (Rolls 2023c) at https://www.oxcns.org/papers/Rolls 2023 Brain Computations and Connectivity.pdf.
